# Antimicrobial resistance in *Clostridioides* (*Clostridium*) *difficile* derived from humans: a systematic review and meta-analysis

**DOI:** 10.1186/s13756-020-00815-5

**Published:** 2020-09-25

**Authors:** Mohammad Sholeh, Marcela Krutova, Mehdi Forouzesh, Sergey Mironov, Nourkhoda Sadeghifard, Leila Molaeipour, Abbas Maleki, Ebrahim Kouhsari

**Affiliations:** Clinical Microbiology Research Center, Ilam University of Medical Sciences, Ilam, Iran

**Keywords:** *Clostridioides difficile*, Antimicrobial resistance, Metronidazole, Vancomycin, Meta-analysis

## Abstract

**Background:**

*Clostridioides* (*Clostridium*) *difficile* is an important pathogen of healthcare- associated diarrhea, however, an increase in the occurrence of *C. difficile* infection (CDI) outside hospital settings has been reported. The accumulation of antimicrobial resistance in *C. difficile* can increase the risk of CDI development and/or its spread. The limited number of antimicrobials for the treatment of CDI is matter of some concern.

**Objectives:**

In order to summarize the data on antimicrobial resistance to *C. difficile* derived from humans, a systematic review and meta-analysis were performed.

**Methods:**

We searched five bibliographic databases: (MEDLINE [PubMed], Scopus, Embase, Cochrane Library and Web of Science) for studies that focused on antimicrobial susceptibility testing in *C. difficile* and were published between 1992 and 2019. The weighted pooled resistance (WPR) for each antimicrobial agent was calculated using a random- effects model.

**Results:**

A total of 111 studies were included. The WPR for metronidazole and vancomycin was 1.0% (95% CI 0–3%) and 1% (95% CI 0–2%) for the breakpoint > 2 mg/L and 0% (95% CI 0%) for breakpoint ≥32 μg/ml. Rifampin and tigecycline had a WPRs of 37.0% (95% CI 18–58%) and 1% (95% CI 0–3%), respectively. The WPRs for the other antimicrobials were as follows: ciprofloxacin 95% (95% CI 85–100%), moxifloxacin 32% (95% CI 25–40%), clindamycin 59% (95% CI 53–65%), amoxicillin/clavulanate 0% (0–0%), piperacillin/tazobactam 0% (0–0%) and ceftriaxone 47% (95% CI 29–65%). Tetracycline had a WPR 20% (95% CI 14–27%) and meropenem showed 0% (95% CI 0–1%); resistance to fidaxomicin was reported in one isolate (0.08%).

**Conclusion:**

Resistance to metronidazole, vancomycin, fidaxomicin, meropenem and piperacillin/tazobactam is reported rarely. From the alternative CDI drug treatments, tigecycline had a lower resistance rate than rifampin. The high-risk antimicrobials for CDI development showed a high level of resistance, the highest was seen in the second generation of fluoroquinolones and clindamycin; amoxicillin/clavulanate showed almost no resistance. Tetracycline resistance was present in one fifth of human clinical *C. difficile* isolates.

## Introduction

*Clostridium difficile,* recently reclassified as *Clostridioides difficile* [1]*,* is an important pathogen of healthcare-associated diarrhea [2]. Recently, however, an increase in the occurrence of CDI outside hospital settings has been reported [3, 4].

Previous antibiotic use was recognized as one of the risk factors for developing CDI through an alteration of gut microbiota. The accumulation of antimicrobial resistance mechanisms may provide an advantage to *C. difficile* as it is not affected by antimicrobials present in the gut [5].

An antibiotic stewardship intervention, that limited the use of the fluoroquinolones, clindamycin, amoxicillin/clavulanate, and cephalosporins, was shown to be effective in reducing the occurrence of multidrug-resistant epidemic ribotypes, e.g. 001 and 027 [6].

Currently, three antimicrobial agents, metronidazole, vancomycin and fidaxomicin are recommended for the treatment of CDI [7–9] and several new anti-CDI drugs are being tested in clinical trials [9]. The new data suggest tigecycline is effective in treating patients with a severe course of CDI [10], and rifaximin might be beneficial in preventing a CDI relapse [11].

In addition to humans, *C. difficile* has been cultured from livestock, food and the environment [12]. Tetracycline is one of the most commonly used antimicrobials in agriculture providing antimicrobial selective pressure in this sphere. This is supported by observations of a high prevalence of the tetracycline resistance gene *tetM* in livestock-associated *C. difficile* ribotype 078 isolates [13]. Moreover, the zoonotic transmission of *C. difficile* between farm animals and humans has been demonstrated [14].

Carbapenems are antimicrobials used for the treatment of infections caused by multidrug-resistant gram-negative pathogens. However, some carbapenem resistance mechanisms are transferable to other bacterial species [15]. Hence, the monitoring of carbapenem resistance in *C. difficile* is justified.

We aimed to review the data on the resistance of antimicrobials to *C. difficile* that have been recommended for CDI treatment; alternative drugs for CDI treatment; high-risk antimicrobials associated with CDI development; agriculture-related antimicrobials; and antimicrobials reserved for the treatment of multidrug pathogens.

## Methods

### Search strategy and study selection

Five bibliographic databases, including international databases (MEDLINE [PubMed], Scopus, Embase, Cochrane Library and Web of Science) were searched for relevant articles (Until October 2019) using the following keywords: (“*Clostridium difficile*” OR “*Clostridioides difficile”* OR *C. difficile*) AND (“Antimicrobial-Drug Resistance” OR “drug resistance” OR “antibiotic resistance” OR “aminoglycosides” OR “beta-lactams” OR “cephalosporins” OR “clindamycin” OR “tetracyclines” OR “fluoroquinolones” OR “macrolides” OR “vancomycin” OR “metronidazole” OR “fidaxomicin” OR “carbapenems”) in the Title/Abstract/Keywords fields. No limitation was used while searching the databases, but for the study to be included in our analysis, the available abstract had to be written in English. The recorded hits were merged, and any duplicates were removed using EndNote X7 (Thomson Reuters, New York, NY, USA).

### Selection criteria and data extraction

All selected studies were reviewed by three authors independently: Ebrahim Kouhsari, Behnam Ahmadzadeh and Abbas Maleki. Studies were excluded if they met the following conditions: (1) *C. difficile* antibiotic resistance was not presented; (2) resistance rates were not clearly reported; (3) no human clinical *C. difficile* strain was tested; (4) it was a meta-analysis and systematic review or a review article or not an original research article; (5) a duplicated report using the same database; (6) a conference abstract and article without the full text upon request from the author; (7) less than 5 isolates were tested. Any discrepancies and inconsistencies with the selection of an article were resolved through discussion, and a fourth author (Nourkhoda Sadeghifard) acted as arbiter.

The information extracted from each included study was: (1) author; (2) publication year; (3) study period; (4) number of *C. difficile* isolates; (5) antimicrobial susceptibility methods; (6) interpretation of resistance; (7) resistance rates (Supplementary Data 1).

### Quality assessment

A quality evaluation of the included studies was performed independently (Behnam Ahmadzadeh, Ebrahim Kouhsari), using an adapted version of the tool proposed by the Newcastle-Ottawa assessment scale adapted for cross-sectional studies [16] (Supplementary Table 1). A score ranging from 0 to 8 points was attributed to each study (≥ 5 points: high quality, 4–3 points: Moderate quality, ≤ 2 points: low quality). A higher score indicated a higher study quality. A third reviewer (Leila Molaeipour) adjudicated in any cases where there was a disagreement.

### Definition of resistance

In individual studies, resistance was defined according to either the European Committee on Antimicrobial Susceptibility Testing (EUCAST) [17] or the Clinical & Laboratory Standards Institute (CLSI) [18] minimal inhibitory concentration (MIC) interpretative breakpoints. The individual MICs were as follows: vancomycin ≥32 mg/L; metronidazole ≥32 mg/L; clindamycin ≥8 mg/L; tetracycline ≥16 mg/L; ciprofloxacin ≥8 mg/L; moxifloxacin ≥8 mg/L; meropenem ≥16 mg/L; piperacillin/tazobactam ≥128/4 mg/L, amoxicillin/clavulanate ≥16/8 mg/L and ceftriaxone 64 mg/L according to the (CLSI) [18]. The MIC interpretive breakpoints for vancomycin >2 mg/L, metronidazole >2 mg/L, rifampin >0.004 mg/L, moxifloxacin >4 mg/L and tigecycline >0.25 mg/L were based on the epidemiological cut-off values (ECOFFs) defined by EUCAST [17].

### Statistical analysis

Studies presenting raw data on antimicrobial resistance were included in the meta-analysis which was performed by computing the pooled prevalence of resistance for each antimicrobial agent using a random- effects model with Stata/SE software, v.14.1 (StataCorp, College Station, TX). The inconsistency across studies was examined by the forest plot as well as the I^2^ statistic. Values of I^2^ (25, 50 and 75%) were interpreted as the presence of low, medium or high heterogeneity, respectively and the random effects models were used [19]. Subgroup analyses were then employed by assuming continents, year, antimicrobial susceptibility testing, and the quality of studies as sources of variation. All statistical interpretations were reported on a 95% confidence interval (CI) basis.

### Study outcomes

The main outcome of interest was the weighted pooled resistance rate (WPR) of strains resistant to specific antimicrobial agents according to the CLSI and/or EUCAST guidelines, respectively. A subgroup analysis was performed (1) for geographical regions (Asia, Europe, Africa, Oceania, South and North America); (2) publication date (1992–2014, and 2015–2019, 3) antimicrobial susceptibility testing method (agar dilution, Etest, and microbroth dilution); and (4) the quality of the studies (high quality, moderate quality, low quality). Subgroup analyses were not performed when the number of studies in the category was lower than five.

## Results

### Search results

**Fig. 1:**
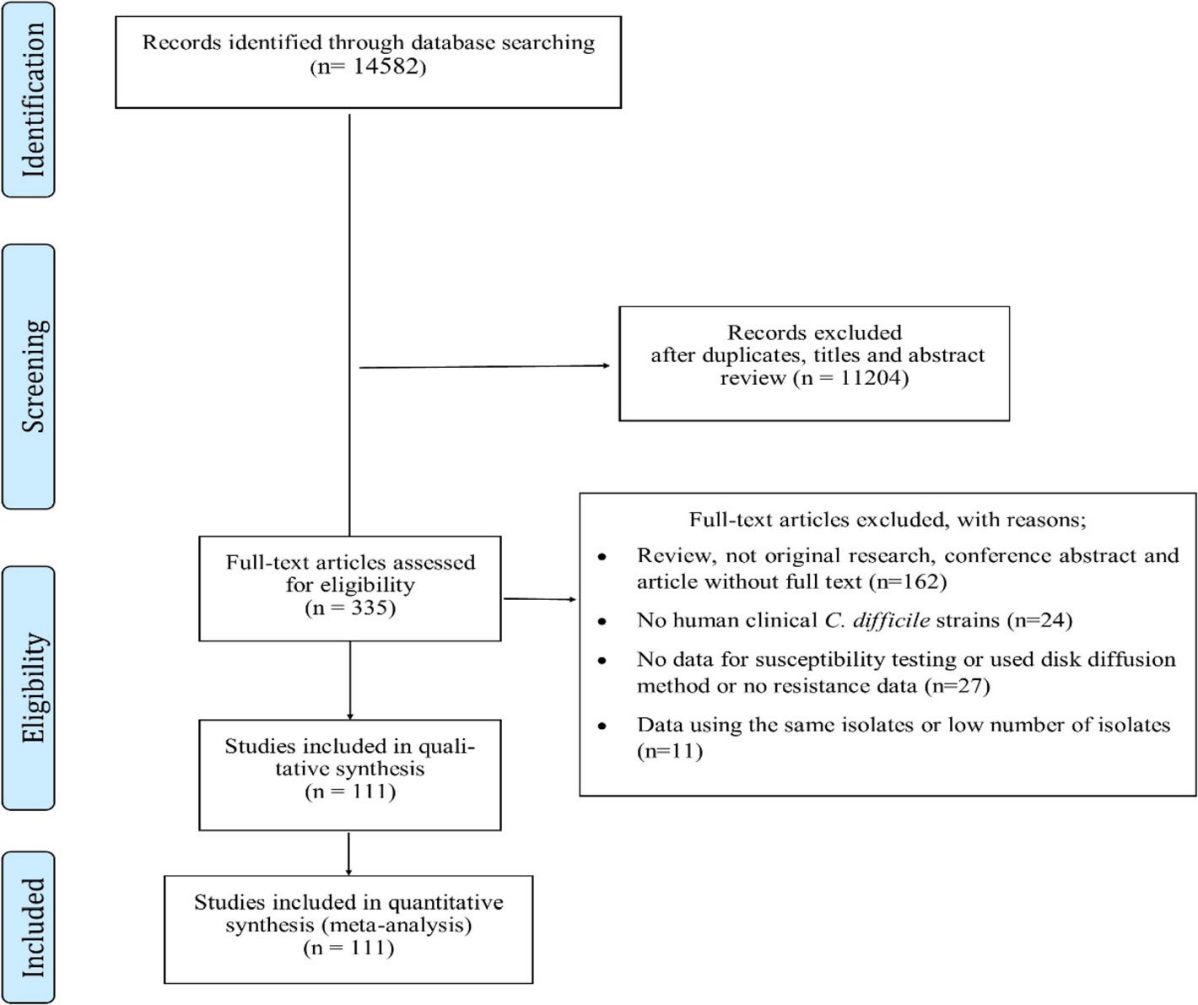
Flow Diagram Showing the Study Selection Process

We evaluated six electronic databases and categorized 14,582 articles published up to October 2019 (Fig. 1). From these, after an initial screening of the title and abstract, 11,204 articles were excluded, due to their irrelevance and duplication, but the full text of the remaining 335 articles was reviewed (Fig. 1). From the 335 articles, 224 were excluded again for the following reasons: review, not original research, conference abstract and article without full text (*n* = 162), no human clinical *C. difficile* strains (*n* = 24), no data for susceptibility testing or used disk diffusion method or no resistance data (*n* = 27), and data using the same isolates or low number of isolates (*n* = 11). Finally, 111 studies were included in this systematic review and meta-analysis (Supplementary Data 1). The studies included in the meta-analysis assessed antibiotic resistance to metronidazole, clindamycin, tetracycline, moxifloxacin and ciprofloxacin, meropenem, piperacillin/tazobactam, amoxicillin/clavulanate, vancomycin, rifampin and tigecycline.

### Characteristics of the included studies

The 111 included studies [20–130] were performed in 35 countries and investigated 19,733 *C. difficile* isolates. The majority of the studies originated in Asia (*n* = 42), followed by Europe (*n* = 37).

Epsilometer (E-test) strips were the most frequent antimicrobial susceptibility testing method used (*n* = 58), followed by agar dilution (*n* = 49). All studies had a cross-sectional design, and the mean Newcastle-Ottawa score was 4.5. The quality was high in 62 (55.8%) studies, medium in 46 (41.4%) studies, and low in 3 (2.7%) studies (Supplementary Data 1). Most of the studies (93.69%) included in the meta-analysis had determined the resistance to metronidazole.

**Fig. 2:**
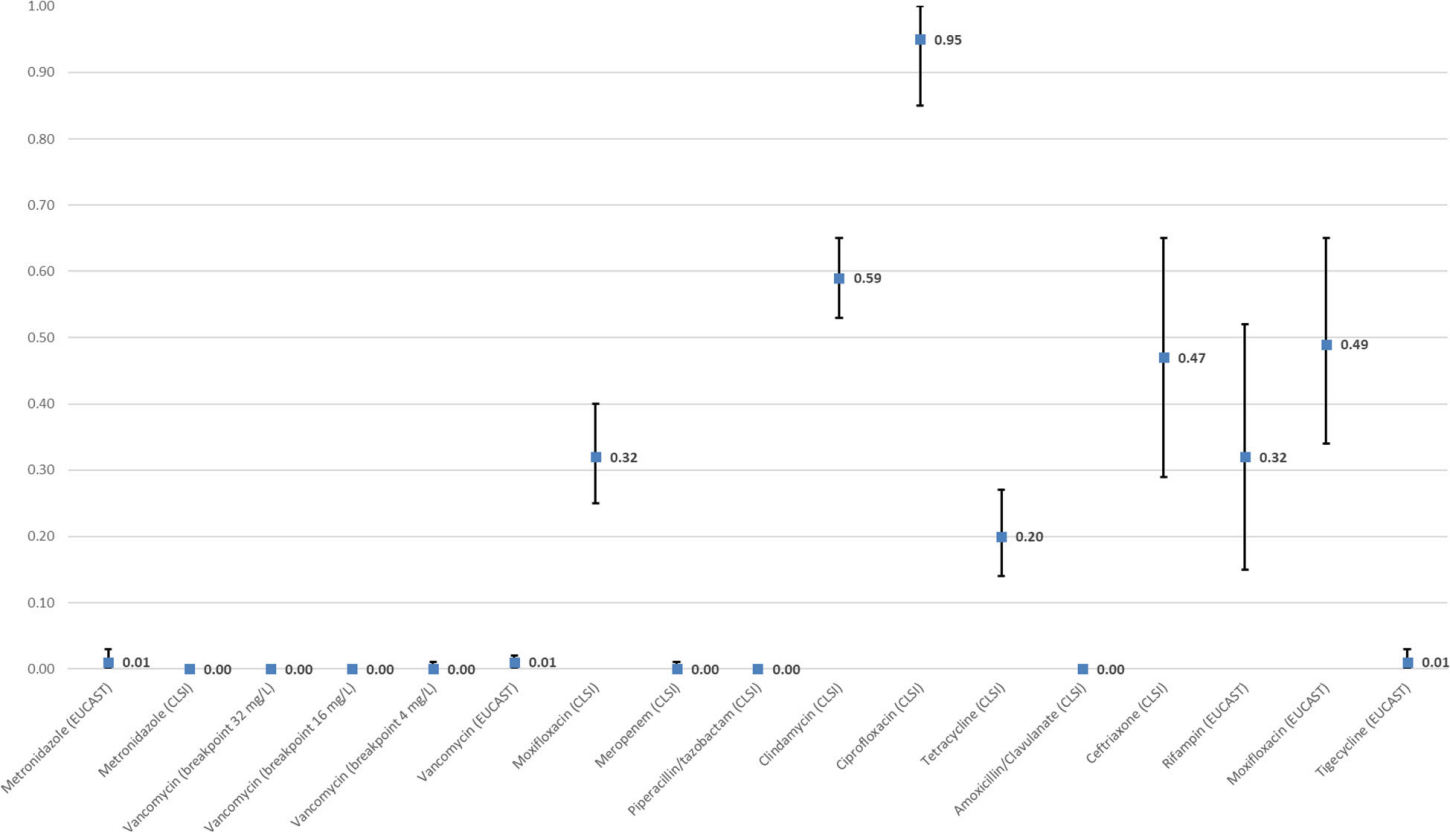
Weighted pooled resistance rate for each antimicrobial in the study

The WPR rates for each antimicrobial are shown in Table 1 and Fig. 2. The forest plots that show the analyses for resistance to individual antimicrobials and subgroups are displayed in the Supplementary Figure 1. Data on the resistance of each antimicrobial and the subgroup analyses by year, continent, quality and method of susceptibility testing are shown in the Supplementary Table 2.

**Table 1:** The WPR rates for each antimicrobial

Antimicrobials	Breakpoint (mg/L)	Number of isolates tested	Number of resistant isolates n (%)	Weighted pooled resistance rate	95% Confidence interval	Heterogeneity
Metronidazole	>2	5900	190 (3.2)	0.01	0–0.03	91.97
Metronidazole	≥32	13,207	129 (1.0)	0.00	0–0.00	81.4
Vancomycin	≥32	2307	13 (0.6)	0.00	0–0.00	38.6
Vancomycin	≥16	2296	10 (0.4)	0.00	0–0.00	7.62
Vancomycin	≥4	1107	7 (0.6)	0.00	0–0.01	50.1
Vancomycin	>2	11,188	416 (3.7)	0.01	0–0.02	89.79
Moxifloxacin	≥8	11,484	3912 (34.1)	0.32	0.25–0.4	93.89
Meropenem	≥16	2756	20 (0.7)	0.00	0–0.01	71.49
Piperacillin/Tazobactam	≥128/4	3041	8 (0.3)	0.00	0–0.00	0
Clindamycin	≥8	19,645	6685 (34.0)	0.59	0.53–0.65	97.50
Ciprofloxacin	≥8	4339	3356 (77.0)	0.95	0.85–1.0	99.12
Tetracycline	≥16	4861	886 (18.2)	0.20	0.14–0.27	97.04
Amoxicillin/Clavulanate	≥16/8	2803	4 (0.1)	0.00	0–0.00	45.4
Ceftriaxone	≥64	3476	1289 (37.1)	0.47	0.29–0.65	99.05
Rifampin	>0.004	1861	787 (42.3)	0.37	0.18–0.58	97.69
Moxifloxacin	>4	2809	929 (33.1)	0.49	0.30–0.67	98.68
Tigecycline	>0.25	2375	39 (1.6)	0.01	0–0.03	83.53

### Resistance to metronidazole

The susceptibility to metronidazole was determined in 104 studies and included 19,645 *C. difficile* isolates.

The EUCAST breakpoint (2 mg/L) was applied in 32 studies (5900 *C. difficile* isolates) and 190 *C. difficile* isolates were found to be resistant (3.2%); the WPR (to metronidazole) was 1% (95% CI 0–3%) with substantial heterogeneity (I^2^ = 91.97%).

The subgroup analysis that compared the data from 1992 to 2014 (WPR 0%; 95% CI 0–1%) and 2015–2019 (WPR 2%; 95% CI 0–4%) indicated an increase in the resistance rate. However, this difference was not statistically significant (*P* = 0.06). Based on the quality of the studies, the resistance rates did not differ between the groups (*p* = 0.998), T\he highest resistance rate was reported in Asia, followed by North America and Europe (4, 95% CI 0–12%; 3, 95% CI 0–8%; 1, 95% CI 0–2%). No statistical difference was found in the method used for antimicrobial susceptibility testing (AST), (*p* = 0.523).

The CLSI breakpoint (32 mg/L) was applied to 69 studies and 129 of the 13,207 *C. difficile* isolates investigated were found to be resistant (1.0%); the WPR was 0% (95% CI 0–0%), with substantial heterogeneity (I^2^ = 81.4%). No statistical significant difference was found between data from 1992 to 2014 and 2015–2019 (*p* = 0.280). Subgroup analyses by continent showed a significant difference between the groups (*p* = 0.038); the highest resistance was in Europe (1%; 95% CI 0–2%). No statistical difference was found in the method used for AST (*P* = 0.281).

### Resistance to vancomycin

A susceptibility to vancomycin was determined in 94 studies where 15,515 *C. difficile* isolates were tested.

Eighteen studies used the breakpoint of 32 mg/L and 13 *C. difficile* isolates of the 2307 isolates tested were resistant (0.6%). Another nine studies used the breakpoint of 16 mg/L and ten *C. difficile* isolates of 2296 tested were resistant (0.4%). Eight studies used the breakpoint of 4 mg/L and seven isolates of the 1107 isolates tested were resistant (0.6%). Overall the WPRs were, based on the breakpoints of 32, 16 and 4 mg/L, 0% (95% CI 0–0%), 0% (95% CI 0–0%), and 0% (95% CI 0–1%) with a heterogeneity of ≤50.1%. The subgroup analyses were not performed.

Based on the EUCAST breakpoint of 2 mg/L, a sensitivity to vancomycin was investigated in 58 studies and of the 11,188 *C. difficile* isolates tested, 416 isolates were found to be resistant (3.7%). The WPR was 1% (95% CI 0–2%), with substantial heterogeneity (I^2^ = 93.89%, *P* = 0.00).

The subgroup analysis, that compared data from 1992 to 2014 (WPR 1%; 95% CI 0–2%) and from 2015 to 2019 (WPR 1%; 95% CI 0–3%), indicated an increase in the resistance rate, however, this difference was not statistically significant (*P* = 0.48). In the continental subgroup analyses, a significant difference was found (*P* = 0.000) and the highest resistance rates were reported in South America followed by North America (53, 95% CI 38–68 and 4%, 95% CI 1–9%).

The resistance rates differ significantly when comparing the quality of studies (*P* = 0.01). In the low quality articles, the WPR was 6% (95% CI 2–11%) higher than in the moderate and high quality articles with a WPR of 2% (95% CI 0–2%). No statistical difference was found in the method used for AST (*P* = 0.47).

### Resistance to moxifloxacin

A susceptibility to moxifloxacin was determined in 78 studies and from those studies 14,383 isolates were investigated.

Using the CLSI breakpoint of 8 mg/L, 11484 *C. difficile* isolates were investigated and 3912 isolates were found to be resistant (34.1%); the WPR to moxifloxacin was 32% (95% CI, 25–40%) with a substantial heterogeneity (I^2^ = 93.89%, *P* = 0.00).

The subgroup analysis, that compared data from 1992 to 2014 and 2015–2019, did not find any significant difference between these groups (*p* = 0.508). In the continental categorisation, the difference between the groups was significant (*P* = 0.000); the highest WPR was in Africa, followed by North America and Asia (94, 95% CI 85–98%; 44, 95% CI 33–55 and 33%, 95% CI 25–40%), respectively. In a subgroup analysis on the quality of articles, the results showed a significant difference (*P =* 0.014); the low, moderate and high quality reports gave WPRs of 54% (95% CI 39–68%), 35% (95% CI 22–49%), and 30% (95% CI 22–38%). No statistical difference was found in the method used for AST (*P* = 0.543).

Using the EUCAST breakpoint of 4 mg/L, 11484 *C. difficile* isolates were investigated and 3912 isolates were found to be resistant; 34.1%); the WPR was 49% (95% CI 0.34–0.65) with a substantial heterogeneity (I^2^ = 98.4%, *P* = 0.00). The subgroup analyses were not performed.

### Resistance to ciprofloxacin

The susceptibility to ciprofloxacin was determined in 28 studies investigating 4339 *C. difficile* isolates and used a breakpoint of 8 mg/L. From them, 3356 isolates were found to be resistant (77%); the WPR to ciprofloxacin was 95% (95% CI 85–100%) with a substantial heterogeneity (I^2^ = 99.12%, *P* = 0.00).

A subgroup analysis, that compared the data from 1992 to 2014 and 2015–2019, showed a significant difference (*P* = 0.001), 100% (95% CI 100%) versus 79% (95% CI 54–97%). The difference in continental categorisation was also significant (*P* = 0.000); the highest WPR was in South America (100, 95% CI 40–100%) followed by Asia (96, 95% CI 89–100% and North America (94, 95% CI 40–100%). No statistical difference was found in the method used for AST (*P* = 0.495).

### Resistance to clindamycin

The susceptibility to clindamycin was determined in 64 studies investigating 19,645 *C. difficile* isolates and, using the CLSI breakpoint (8 mg/l), 6685 *C. difficile* isolates were reported to be resistant (34.0%).

The overall WPR to clindamycin was 59% (95% CI, 53–65%), with a substantial heterogeneity (I^2^ = 97.50%, *P* = 0.00); there was no significant difference in the time categories (*P* = 0.96). The groups differed in continental categorization (*p* = 0.000) with the highest rates in Asia and South America (72, 95% CI 65–78 and 59%, 95% CI 19–94%, respectively). Also, in the subgroup analysis on the quality of articles, the results showed a significant difference (*P =* 0.000); the low, moderate and high quality reports reported resistance rates of 17% (95%CI 9–27%), 57% (95% CI 46–68%) and 63% (95% CI 55–70%), respectively. There was statistical significance between the methods used for AST (*p* = 0.020).

### Resistance to tetracycline

The susceptibility to tetracycline was determined in 31 studies investigating 4861 *C. difficile* isolates and from those 886 isolates (18.2%) were found to be resistant using the breakpoint of 16 mg/L. The WPR was 20% (95% CI, 14–27%), with substantial heterogeneity (I^2^ = 97.04%, *P* = 0.00).

There was no difference between the data from 1992 to 2014 and 2015–2019 (*p* = 0.26). A statistically significant difference was found in the continental categorization (*P* = 0.000); the highest resistances were 34% (95% CI, 26–43%), 26% (95% CI, 17–35%), and 16% (95% CI, 5–31%) in Oceania, Asia, and Europe, respectively. In a subgroup analysis on the quality of articles, the results showed a significant difference (*P =* 0.01); the low, moderate and high quality reports gave resistance rates of 40% (95%CI 29–52%), 16% (95% CI 7–28%) and 22% (95% CI 13–32%), respectively. No statistical difference was found in the method used for AST (*P* = 0.216).

### Meropenem

The susceptibility to meropenem was determined in 17 studies using the breakpoint (≥16 mg/L mg/L) and 2756 *C. difficile* isolates were investigated; 20 isolates found to be resistant (0.7%). The overall WPR was 0% (95% CI, 0%-%1) with moderate heterogeneity (I^2^ = 71.49%, *P* = 0.00). No statistical difference was found between the data from 1992 to 2014 and 2015–2019 (*p* = 0.106). The continental, quality and methods subgroup differences were not analysed.

### Amoxicillin/Clavulanate

The susceptibility to co-amoxicillin was investigated in 10 studies using the breakpoint of ≥16/8 mg/L. A total of 2803 *C. difficile* isolates were investigated and 4 isolates were reported as resistant (0.1%); the WPR was 0% (95% CI, 0–0%), with low heterogeneity (I^2^ = 45.4%, *P* = 0.06). No subgroup analyses were performed.

### Piperacillin/Tazobactam

The susceptibility to piperacillin/tazobactam was investigated in 17 studies applying the breakpoint of ≥128/4 mg/L mg/L and included 3041 *C. difficile* isolates. Eight isolates were found to be resistant (0.3%); the WPR to this antibiotic was 0% with (95% CI, 0–0%). No subgroup analyses were performed.

### Ceftriaxone

The susceptibility to ceftriaxone was investigated in 13 studies. Of the 3476 *C. difficile* isolates investigated, 1289 isolates were found to be resistant (37.1%) using the breakpoint of ≥64 mg/L. The WPR for ceftriaxone was 47% (95% CI, 29–65%), with substantial heterogeneity (I^2^ = 99.05%, *P* = 0.00). No subgroup analyses were performed.

### Rifampin

The susceptibility to rifampin was investigated in 10 studies on 1861 of *C. difficile* isolates. Using the breakpoint of 0.004 mg/L, 787 isolates were reported to be resistant (42.3%), the WPR was 37% (95% CI, 18–58%) with substantial heterogeneity (I^2^ = 97.69%, *P* = 0.00). No subgroup analyses were performed.

### Tigecycline

The susceptibility to tigecycline was investigated in 10 studies in 2375 *C. difficile* isolates. Thirty-nine isolates were reported to be resistant (1.6%) based on the breakpoint of 0.25 mg/L; the WPR was 1% (95% CI 0–3%) with substantial heterogeneity (I^2^ = 83.53%, *P* = 0.00). No subgroup analyses were performed.

### Fidaxomicin

The susceptibility to fidaxomicin was investigated in 1184 isolates from six studies. One isolate found to be resistant (0.08%) based on the breakpoint of ≥8 mg/L. The analyses were not performed because of the absence of a recommended breakpoint and the low number of studies.

## Discussion

Due to the limited number of antimicrobials that can be used for the treatment [7–9] of CDI, it is important to obtain information about the resistance profiles of circulating *C. difficile* strains. Moreover, the accumulation of resistance mechanisms gives *C. difficile* an advantage since CDI can develop after the use of antimicrobials due to an alteration in gut microbiota [5].

Several methods can be used to determine the MIC in antimicrobial susceptibility testing. In our study, the Etest was the most used method followed by agar dilution. The agar dilution method is suitable for AST when there is high number of isolates since there is a need to prepare fresh testing plates for each experiment; however the commercially available Etest can be used independently for individual isolates.

Three antimicrobials are recommended for the treatment of CDI; metronidazole, vancomycin and fidaxomicin. For AST, there is still no MIC breakpoint available for fidaxomicin; for vancomycin and metronidazole two values exist but with a wide range: EUCAST 2 mg/L and CLSI 32 mg/L, The difference between the resistance rates, according to the breakpoint used, was also noted in our study. For metronidazole, the WPR was 1% (95% CI, 0–3%) using EUCAST but using CLSI, the WPR was 0% (95% CI, 0–0%). A similar pattern was also observed for vancomycin where using the EUCAST breakpoint, the WPR was higher (1% (95% CI 0–2%) than for the CLSI breakpoint 0% (95% CI, 0–0%).

Recently, a systematic review and meta-analysis [131] of metronidazole and vancomycin resistance in *C. difficile* showed higher WPRs than observed in our study; 1.9% (95% CI, 0.5–3.6%) for metronidazole and 2.1% (95% CI, 0–5.1%) for vancomycin. The analyses differed in the date of publication for data collection, (1982–2017) vs (1992–2019), and in the origin of the isolates since, in our analyses, the data on the *C. difficile* isolates of animal origin were not included.

The data on the susceptibility testing for metronidazole, vancomycin and moxifloxacin were included in the “enhanced level” of a CDI surveillance protocol published by the European Centre for Disease Prevention and Control (ECDC) [2]. Moxifloxacin, a fluoroquinolone, is not considered as a drug for CDI treatment but moxifloxacin resistance in *C. difficile* strains was shown to be an important marker for the spread of *C. difficile* in a healthcare setting [132]. Two representatives of fluoroquinolones were analysed in our study: ciprofloxacin and moxifloxacin. From all the antimicrobials in our study, ciprofloxacin showed the highest level of resistance (WPR 95%) and the resistance to moxifloxacin was 32 and 49% according to the CLSI and EUCAST breakpoints, respectively.

In addition to fluoroquinolones, clindamycin, amoxicillin/clavulanate and cephalosporins are indicated for limited use in hospital settings in order to reduce CDI rates [6]. From these four classes of antimicrobials, three classes exhibited high rates of resistance; however with amoxicillin/clavulanate, only 4 isolates out of 2803 isolates were investigated.

Rifaximin has been suggested as an alternative to existing CDI therapies, especially in CDI recurrences and their prevention [133, 134]. Data on rifampin resistance, which correlate with rifaximin [135], showed a high level of resistance in investigated *C. difficile* isolates (787/1861) and suggest more risk to treatment failure due to *C. difficile* strain resistance compared to recommended CDI treatments.

The effectiveness of tigecycline use in the treatment of CDI was evaluated in several studies [10]. According to the reported resistance rates in our study, treatment failure is less likely with tigecycline than with rifaximin. However, recently, the emergence of mobile tigecycline-resistance genes, *tet(*X3) and *tet*(X4) that inactivate all tetracyclines, including tigecycline, was reported recently in gram-negative bacteria [136]. Moreover, Tet proteins have, in vitro*,* the potential to acquire mutations leading to an increased MICs for tigecycline [137]. From the available data, the *tet* classes of ribosomal protection genes are the most common molecular mechanism for tetracycline resistance in *C. difficile* [13]. The spread of newly detected *tet*(X) genes or mutations in present *tet* classes genes (e.g *tetM* or *tetW*) could increase the prevalence of resistance to tigecycline.

## Conclusion

A resistance to metronidazole, vancomycin, fidaxomicin, meropenem and piperacillin-tazobactam is reported rarely. From alternative CDI treatment drugs, tigecycline had a lower resistance rate than rifampicin. The high-risk antimicrobials for CDI development showed a high level of resistance, the highest was seen in the second generation of fluoroquinolones and clindamycin; amoxicillin/clavulanate showed almost no resistance. Tetracycline resistance was present in one fifth of human clinical *C. difficile* isolates.

## Supplementary information

**Additional file 1.**

**Additional file 2.**

**Additional file 3.**

**Additional file 4.**

## Data Availability

All data were included.

## Abbreviations

WPR: Weighted Pooled Resistance
CDI: *Clostridium difficile* infection
EUCAST: European Committee on Antimicrobial Susceptibility Testing
CLSI: Clinical & Laboratory Standards Institute
MIC: Minimal inhibitory concentration
ECOFFs: Epidemiological cut-off values
AST: Antimicrobial susceptibility testing
ECDC: European Centre for Disease Prevention and Control
