# Supplementary material for: Antimicrobial resistance in Clostridioides (Clostridium) difficile derived from humans: a systematic review and meta-analysis
Source: Antimicrob Resist Infect Control. 2020 Sep 25;9:158. doi: 10.1186/s13756-020-00815-5 (PMC7517813; doi:10.1186/s13756-020-00815-5)
Supplement: Supplementary file 1 — Additional file 1. [file 13756_2020_815_MOESM1_ESM.docx]

Supplementary Data 1. Characteristics of included studies.

| ID | Authors | Year | Literature Quality | Country | Testing Method | No. of isolates | Metronidazole Resistance No, Breakpoint | Vancomycin Resistance No, Breakpoint | Moxifloxacin Resistance No | Meropenem Resistance No | Piperacillin Tazobactam Resistance No, | Clindamycin Resistance No | Ciprofloxacin Resistance No | Tetracycline Resistance No | Amoxicillin Clavulanate Resistance No | Ceftriaxone Resistance No | Metronidazole Resistance No, Breakpoint | Vancomycin Resistance No Breakpoint | Rifampin Resistance No | Moxifloxacin Resistance No | Tigecycline Resistance No | Fidaxomicin Resistance No |
| --- | --- | --- | --- | --- | --- | --- | --- | --- | --- | --- | --- | --- | --- | --- | --- | --- | --- | --- | --- | --- | --- | --- |
|  |  |  |  |  |  |  | CLSI Breakpoint | | | | | | | | | | EUCAST Breakpoint | | | | | |
| 1 | Ackermann et al ([1](#_ENREF_1)) | 2003 | 4 | Germany | ET | 192 | 0, 8 | 0, 16 |  |  |  | 69 |  |  |  |  | 0, 8 | 0, 16 |  | 23 |  |  |
| 2 | Ackermann et al ([2](#_ENREF_2)) | 2004 | 4 | Germany | AD | 207 | 0, 32 | 0, 16 | 26 |  |  |  |  |  |  |  | 0, 32 | 0, 16 |  |  |  |  |
| 3 | Álvarez-Pérez et al ([3](#_ENREF_3)) | 2017 | 2 | Spain | ET | 50 | 2, 32 | 0, 32 | 27 |  |  | 10 |  | 32 |  |  | 2, 32 | 0, 32 |  |  |  |  |
| 4 | Androga et al ([4](#_ENREF_4)) | 2018 | 4 | Australia | AD | 28 | 0, 2 | 0, 2 | 3 | 0 |  | 3 |  | 3 | 0 |  | 0, 2 | 0, 2 |  |  |  |  |
| 5 | Aoki et al ([5](#_ENREF_5)) | 2019 | 5 | Japan | AD | 188 | 0, 32 | 0, 4 | 124 |  |  | 172 |  |  |  |  | 0, 32 | 0, 4 |  |  |  |  |
| 6 | Aptekorz et al ([6](#_ENREF_6)) | 2017 | 3 | Poland | ET | 89 | 0, 2 | 0, 2 |  |  |  |  |  |  |  |  | 0, 2 | 0, 2 | 28 | 89 |  |  |
| 7 | Arca-Suárez et al ([7](#_ENREF_7)) | 2018 | 4 | Spain | ET | 70 | 1, 32 | 0, 2 | 15 |  |  |  |  |  |  |  | 1, 32 | 0, 2 |  |  |  |  |
| 8 | Baghani et al ([8](#_ENREF_8)) | 2018 | 5 | Iran | ET | 46 | 31, 2 | 0, 2 |  |  |  |  |  |  |  |  | 31, 2 | 0, 2 |  | 36 |  |  |
| 9 | Balassiano et al ([9](#_ENREF_9)) | 2011 | 4 | USA | ET | 8 | 0, 32 | 0, 8 |  |  |  | 8 | 8 |  |  |  | 0, 32 | 0, 8 |  |  |  |  |
| 10 | Beran et al ([10](#_ENREF_10)) | 2014 | 6 | Czech Republic | ET | 62 | 0, 2 |  |  |  |  |  |  |  |  |  | 0, 2 | 0, 2 |  | 42 |  |  |
| 11 | Beran et al ([11](#_ENREF_11)) | 2017 | 6 | Czech Republic | AD | 64 | 0, 2 | 0, 2 |  |  |  |  |  |  |  |  | 0, 2 | 0, 2 |  | 43 |  |  |
| 12 | Berger et al ([12](#_ENREF_12)) | 2018 | 4 | Lebanon | ET | 107 | 0, 2 | 0, 2 |  |  |  |  |  |  |  |  | 0, 2 | 0, 2 |  | 6 |  |  |
| 13 | Bourgault et al ([13](#_ENREF_13)) | 2006 | 4 | Canada | ET | 258 | 0, 32 | 0, 32 | 212 | 0 | 1 | 38 | 258 |  |  | 213 | 0, 32 | 0, 32 |  |  |  |  |
| 14 | Büchler et al ([14](#_ENREF_14)) | 2014 | 5 | Switzerland | ET | 86 | 0, 32 | 1, 2 |  | 0 | 0 | 45 | 85 |  | 4 | 33 | 0, 32 | 1, 2 |  |  |  |  |
| 15 | Byun et al ([15](#_ENREF_15)) | 2019 | 6 | South Korea | AD | 331 | 0, 2 | 0, 2 |  |  |  |  |  |  |  |  | 0, 2 | 0, 2 |  |  |  |  |
| 16 | Chatedaki et al ([16](#_ENREF_16)) | 2019 | 4 | Greece | ET | 88 | 0, 2 | 0, 2 |  |  |  | 54 |  | 21 |  |  | 0, 2 | 0, 2 | 32 | 63 |  |  |
| 17 | Chen et al ([17](#_ENREF_17)) | 2018 | 4 | China | ET | 405 | 0, 32 | 0, 2 | 112 |  |  | 293 | 405 | 87 |  |  | 0, 32 | 0, 2 |  |  |  |  |
| 18 | Cheng et al ([18](#_ENREF_18)) | 2017 | 6 | China | AD | 101 | 0, 32 | 0, 2 | 35 |  |  |  |  |  |  |  | 0, 32 | 0, 2 |  |  |  |  |
| 19 | Chia et al ([19](#_ENREF_19)) | 2013 | 4 | Taiwan | AD | 110 | 2, 2 | 5, 2 |  |  |  |  |  |  |  |  | 2, 2 | 5, 2 |  |  |  |  |
| 20 | Chow et al ([20](#_ENREF_20)) | 2017 | 3 | China | AD | 284 | 0, 32 | 4, 2 | 65 | 0 | 0 | 238 |  | 85 |  |  | 0, 32 | 4, 2 |  |  |  |  |
| 21 | Costa et al ([21](#_ENREF_21)) | 2017 | 5 | Brazil | ET | 4 | 0, 32 | 0, 2 | 0 |  |  | 1 | 4 |  |  |  | 0, 32 | 0, 2 |  |  |  |  |
| 22 | Dong et al ([22](#_ENREF_22)) | 2013 | 4 | China | AD | 60 | 0, 32 | 0, 32 | 18 |  |  | 44 | 60 | 25 |  |  | 0, 32 | 0, 32 |  |  |  |  |
| 23 | Dong et al ([23](#_ENREF_23)) | 2014 | 6 | China | AD | 94 | 0, 32 | 0, 32 | 31 |  |  | 65 | 94 | 32 |  |  | 0, 32 | 0, 32 |  |  |  |  |
| 24 | Ebrahim-Saraie et al ([24](#_ENREF_24)) | 2018 | 5 | Iran | AD | 45 | 0, 32 | 0, 2 |  |  |  |  |  |  |  |  | 0, 32 | 0, 2 |  |  |  |  |
| 25 | Eckert et al ([25](#_ENREF_25)) | 2013 | 4 | France | ET | 224 | 0, 32 | 0, 16 | 18 |  |  | 78 |  | 0 |  |  | 0, 32 | 0, 16 |  |  |  |  |
| 26 | Eitel et al ([26](#_ENREF_26)) | 2014 | 3 | Hungary | ET | 188 | 0, 32 | 0, 2 |  |  |  |  |  |  |  |  | 0, 32 | 0, 2 |  |  |  |  |
| 27 | Fenner et al ([27](#_ENREF_27)) | 2008 | 5 | Switzerland | ET | 124 | 0, 8 | 0, 2 | 28 |  |  | 34 |  |  |  |  | 0, 8 | 0, 2 |  |  |  |  |
| 28 | Fraga et al ([28](#_ENREF_28)) | 2016 | 5 | Brazil | AD | 50 | 0, 2 | 29, 2 | 4 |  |  |  |  |  |  |  | 0, 2 | 29, 2 |  |  | 1 |  |
| 29 | Freeman et al ([29](#_ENREF_29)) | 2014 | 5 | UK | AD | 918 | 1, 8 | 8, 8 | 367 |  |  | 455 |  |  |  |  | 1, 8 | 8, 8 |  |  | 4 |  |
| 30 | Gao et al ([30](#_ENREF_30)) | 2016 | 5 | china | AD | 162 | 0, 2 | 0, 2 | 73 |  | 0 | 129 |  | 76 |  |  | 0, 2 | 0, 2 |  |  |  |  |
| 31 | Giufrè et al ([31](#_ENREF_31)) | 2018 | 3 | Italy | AD | 21 | 12, 2 | 13, 2 | 14 |  |  | 12 |  |  |  |  | 12, 2 | 13, 2 |  |  |  |  |
| 32 | Goudarzi et al ([32](#_ENREF_32)) | 2013 | 5 | Iran | AD | 75 | 4, 32 | 6, 3 |  |  |  | 67 |  |  |  |  | 4, 32 | 6, 3 |  |  |  |  |
| 33 | Harvala et al ([33](#_ENREF_33)) | 2016 | 3 | Sweden | ET | 51 | 0, 2 | 0, 2 |  |  |  |  |  |  |  |  | 0, 2 | 0, 2 |  | 48 |  |  |
| 34 | Hastey et al ([34](#_ENREF_34)) | 2017 | 3 | USA | AD | 920 | 1, 32 | 28, 2 |  |  |  |  |  |  |  |  | 80, 2 | 28, 2 |  |  |  |  |
| 35 | Hecht et al ([35](#_ENREF_35)) | 2007 | 4 | USA | AD | 110 | 0, 32 | 1, 4 | 15 |  |  |  |  |  |  |  | 0, 32 | 1, 4 |  |  |  |  |
| 36 | hidalgo-Villeda et al ([36](#_ENREF_36)) | 2018 | 4 | Honduras | ET | 10 | 0, 32 |  | 10 | 0 |  |  |  | 2 |  |  | 0, 32 | , 4 | 2 |  | 3 |  |
| 37 | Huang et al ([37](#_ENREF_37)) | 2009 | 6 | China | AD | 56 | 0, 32 | 0, 32 | 26 | 0 | 0 | 40 | 56 | 20 |  |  | 0, 32 | 0, 32 |  |  |  |  |
| 38 | Huang et al ([38](#_ENREF_38)) | 2010 | 5 | China | AD | 110 | 0, 32 | 0, 32 | 68 |  | 0 | 97 | 110 | 69 |  |  | 0, 32 | 0, 32 |  |  |  |  |
| 39 | Hung et al ([39](#_ENREF_39)) | 2018 | 5 | Taiwan | AD | 1112 | 14, 32 | 10, 16 |  |  |  |  |  |  |  |  | 14, 32 | 10, 16 |  |  |  |  |
| 40 | Jamal et al ([40](#_ENREF_40)) | 2002 | 5 | Kuwait | ET | 73 |  | 0, 4 |  |  |  |  |  |  | 0 |  | , 8 | 0, 4 |  |  |  |  |
| 41 | Jamal et al ([41](#_ENREF_41)) | 2016 | 5 | Kuwait | ET | 146 | 3, 32 | 0, 2 |  | 15 | 0 | 68 |  |  | 0 |  | 3, 32 | 0, 2 | 17 |  | 17 |  |
| 42 | Jiang et al ([42](#_ENREF_42)) | 2010 | 4 | USA | ET | 359 |  |  |  |  |  |  |  |  |  |  |  |  |  |  |  |  |
| 43 | Jiménez et al ([43](#_ENREF_43)) | 2018 | 4 | Costa Rica | ET | 76 | 0, 16 | 0, 4 |  |  |  | 16 | 32 |  |  |  | 0, 16 | 0, 4 |  | 23 |  |  |
| 44 | Jin et al ([44](#_ENREF_44)) | 2017 | 5 | China | ET | 411 | 64, 32 | 0, 32 | 104 |  | 5 | 257 | 334 | 149 |  |  | 64, 32 | 0, 32 | 38 |  |  |  |
| 45 | John et al ([45](#_ENREF_45)) | 2005 | 5 | UK | ET | 271 |  |  |  |  | 0 |  | 271 |  |  |  |  |  |  |  |  |  |
| 46 | Karlowsky et al ([46](#_ENREF_46)) | 2012 | 4 | Canada | AD | 432 | 0, 32 | 16, 2 | 195 | 0 | 0 | 218 |  |  | 0 | 153 | 0, 32 | 16, 2 |  |  |  |  |
| 47 | Karlowsky et al ([47](#_ENREF_47)) | 2018 | 6 | Canada | AD | 1310 | 3, 2 | 1, 2 | 395 |  |  | 833 |  |  | 0 | 368 | 3, 2 | 1, 2 |  |  |  |  |
| 48 | Kim et al ([48](#_ENREF_48)) | 2012 | 5 | South Korea | ET | 131 | 0, 32 | 0, 32 | 82 |  | 0 | 89 |  |  |  |  | 0, 32 | 0, 32 |  |  |  |  |
| 49 | Knight et al ([49](#_ENREF_49)) | 2015 | 6 | Australia | AD | 440 | 0, 32 | 0, 2 | 15 | 0 |  | 371 |  |  | 0 | 80 | 0, 32 | 0, 2 |  |  |  | 0 |
| 50 | Knight et al ([50](#_ENREF_50)) | 2016 | 5 | Australia | AD | 91 | 0, 2 | 0, 2 | 15 | 0 | 0 |  |  | 39 | 0 | 2 | 0, 2 | 0, 2 |  |  |  |  |
| 51 | Kociolek et al ([51](#_ENREF_51)) | 2016 | 6 | USA | AD | 196 | 0, 32 | 6, 4 | 3 |  |  | 42 |  |  |  |  | 0, 32 | 6, 4 |  |  |  |  |
| 52 | Kouhsari et al ([52](#_ENREF_52)) | 2019 | 5 | Iran | ET | 35 | 2, 32 | 3, 2 | 7 |  |  | 18 |  | 5 |  |  | 2, 32 | 3, 2 |  |  |  |  |
| 53 | Krutova et al ([53](#_ENREF_53)) | 2015 | 2 | Czech Republic | ET | 20 | 0, 2 | 0, 2 |  |  |  | 2 | 20 | 0 |  |  | 0, 2 | 0, 2 | 13 | 20 | 0 |  |
| 54 | Kullin et al ([54](#_ENREF_54)) | 2018 | 3 | South Africa | ET | 77 | 0, 32 | 0, 16 | 72 |  |  |  |  |  |  |  | 0, 32 | 0, 16 |  |  |  |  |
| 55 | Kuwata et al ([55](#_ENREF_55)) | 2014 | 6 | Japan | AD | 130 | 0, 32 | 0, 32 |  | 0 |  | 59 |  |  |  | 49 | 0, 32 | 0, 32 |  |  |  |  |
| 56 | Lachowicz et al ([56](#_ENREF_56)) | 2014 | 5 | Poland | ET | 83 | 0, 2 | 0, 2 |  |  |  | 23 | 83 | 2 |  |  | 0, 2 | 0, 2 | 15 | 69 | 0 |  |
| 57 | Li et al ([57](#_ENREF_57)) | 2019 | 5 | China | ET | 319 | 0, 32 | 0, 4 | 76 |  |  | 226 | 231 | 19 |  |  | 0, 32 | 0, 4 |  |  |  |  |
| 58 | Liao et al ([58](#_ENREF_58)) | 2012 | 5 | Taiwan | AD | 403 | 0, 32 | 2, 2 | 72 |  |  | 296 |  |  |  |  | 0, 32 | 2, 2 |  |  |  |  |
| 59 | Lidan et al ([59](#_ENREF_59)) | 2016 | 4 | China | ET | 9 | 0, 32 | 0, 2 | 2 |  |  | 9 |  |  |  |  | 0, 32 | 0, 2 |  |  |  |  |
| 60 | Lopez-Urena et al ([60](#_ENREF_60)) | 2014 | 3 | Panama | ET | 6 | 0, 32 |  |  |  |  | 6 | 6 |  |  |  | 0, 32 | 0, 32 |  |  |  |  |
| 61 | López-Ureña et al ([61](#_ENREF_61)) | 2016 | 4 | Costa Rica | ET | 68 | 0, 32 | 0, 16 | 34 |  |  | 60 | 68 |  |  |  | 0, 32 | 0, 16 |  |  |  |  |
| 62 | Luo et al ([62](#_ENREF_62)) | 2018 | 6 | China | AD | 85 | 0, 32 | 0, 32 |  |  |  | 50 |  | 30 |  |  | 0, 32 | 0, 32 |  |  |  |  |
| 63 | Mutlu et al ([63](#_ENREF_63)) | 2007 | 5 | UK | AD | 116 | 0, 8 | 0, 8 | 101 |  |  | 73 |  | 5 |  | 101 | 0, 8 | 0, 8 |  |  |  |  |
| 64 | Nasereddin et al ([64](#_ENREF_64)) | 2009 | 5 | Jordan | AD | 41 | 0, 32 | 0, 32 |  |  |  |  |  |  |  |  | 0, 32 | 0, 32 |  |  |  |  |
| 65 | Ngamskulrungroj et al ([65](#_ENREF_65)) | 2015 | 5 | Thailand | ET | 53 | 0, 2 | 1, 2 |  |  |  |  |  |  |  |  | 0, 2 | 1, 2 |  | 24 | 0 |  |
| 66 | Niyogi et al ([66](#_ENREF_66)) | 1992 | 4 | India | AD | 43 | 0, 32 |  |  |  |  |  | 43 | 5 |  |  | 0, 32 |  |  |  |  |  |
| 67 | Novak et al ([67](#_ENREF_67)) | 2014 | 4 | Italy | ET | 50 | 0, 32 | 0, 16 |  |  |  |  |  |  |  |  | 0, 32 | 0, 16 |  |  |  |  |
| 68 | Obuch-Woszczatyński et al ([68](#_ENREF_68)) | 2013 | 3 | Poland | ET | 10 | 0, 1 | 0, 2 |  |  |  | 7 | 10 |  |  |  | 0, 1 | 0, 2 |  | 8 |  |  |
| 69 | Oka et al ([69](#_ENREF_69)) | 2011 | 4 | Japan | ET | 73 | 0, 32 | 0, 32 |  |  |  | 64 |  |  |  | 68 | 0, 32 | 0, 32 |  |  |  |  |
| 70 | Peláez et al ([70](#_ENREF_70)) | 2002 | 6 | Spain | AD | 415 | 26, 32 | 13, 32 |  |  |  |  |  |  |  |  | 26, 32 | 13, 32 |  |  |  |  |
| 71 | Peng et al ([71](#_ENREF_71)) | 2017 | 5 | USA | BM | 139 | 0, 32 |  | 40 |  |  | 115 |  |  |  | 15 | 0, 32 | , 8 |  |  |  | 1 |
| 72 | Peretz et al ([72](#_ENREF_72)) | 2016 | 5 | Israel | ET | 29 | 3, 2 | 5, 2 |  |  |  |  |  |  |  |  | 3, 2 | 5, 2 |  |  |  |  |
| 73 | Piepenbrock et al ([73](#_ENREF_73)) | 2019 | 5 | Germany | AD | 160 | 1, 2 | 0, 2 |  |  |  |  |  |  |  |  | 1, 2 | 0, 2 |  |  |  |  |
| 74 | Pinto et al ([74](#_ENREF_74)) | 2003 | 6 | Brazil | ET | 65 | 0, 32 | 0, 4 |  |  |  | 30 |  |  |  |  | 0, 32 | 0, 4 |  |  |  |  |
| 75 | Pirs et al ([75](#_ENREF_75)) | 2013 | 3 | Slovenia | BM | 92 | 0, 2 | 0, 2 |  | 0 |  | 39 |  | 11 | 0 |  | 0, 2 | 0, 2 |  | 11 |  |  |
| 76 | Putsathit et al ([76](#_ENREF_76)) | 2017 | 4 | Thailand | AD | 105 | 0, 2 | 0, 2 | 22 | 0 |  | 70 |  |  | 0 |  | 0, 2 | 0, 2 |  |  |  |  |
| 77 | Ramírez-Vargas et al ([77](#_ENREF_77)) | 2017 | 3 | UK | ET | 38 | 0, 2 | 4, 2 | 0 |  |  | 0 | 0 | 33 |  |  | 0, 2 | 4, 2 |  |  |  |  |
| 78 | Reil et al ([78](#_ENREF_78)) | 2012 | 5 | Germany | ET | 34 |  |  |  |  |  |  |  |  |  |  |  |  |  | 24 |  |  |
| 79 | Roberts et al ([79](#_ENREF_79)) | 2011 | 4 | New Zealand | AD | 101 | 0, 32 |  | 2 | 0 | 0 | 62 |  |  |  |  | 0, 32 |  |  |  |  |  |
| 80 | Rodriguez et al ([80](#_ENREF_80)) | 2015 | 3 | Belgium | ET | 13 | 0, 32 |  |  |  |  |  |  |  |  |  | 0, 32 | 0, 2 |  |  |  |  |
| 81 | Russello et al ([81](#_ENREF_81)) | 2012 | 4 | Italy | ET | 21 | 0, 32 | 0, 32 |  |  |  |  |  |  |  |  | 0, 32 | 0, 32 |  | 13 |  |  |
| 82 | Saatian et al ([82](#_ENREF_82)) | 2010 | 5 | USA | ET | 41 | 0, 32 |  |  |  |  |  |  |  |  |  | 0, 32 | 0, 2 |  |  |  |  |
| 83 | Samonis et al ([83](#_ENREF_83)) | 2016 | 5 | Greece | ET | 263 | 8, 32 | 0, 2 |  |  |  | 226 |  | 47 |  |  | 8, 32 | 0, 2 |  |  |  |  |
| 84 | Sandell et al ([84](#_ENREF_84)) | 2016 | 4 |  | AD | 81 | 0, 2 | 0, 2 |  |  |  | 59 |  |  |  |  | 0, 2 | 0, 2 |  | 10 | 0 | 0 |
| 85 | Santos et al ([85](#_ENREF_85)) | 2016 | 6 | Portugal | ET | 457 | 16, 2 | 2, 2 |  |  |  |  |  |  |  |  | 16, 2 | 2, 2 |  | 2 |  |  |
| 86 | Secco et al ([86](#_ENREF_86)) | 2014 | 4 | Brazil | ET | 4 | 0, 32 | 0, 8 |  |  |  | 4 |  |  |  |  | 0, 32 | 0, 8 |  | 3 |  |  |
| 87 | Seo et al ([87](#_ENREF_87)) | 2018 | 5 | South Korea | AD | 207 | 0, 32 | 3, 2 |  |  | 0 | 202 |  |  |  |  | 0, 32 | 3, 2 |  |  |  |  |
| 88 | Seugendo et al ([88](#_ENREF_88)) | 2015 | 4 | Tanzania | ET | 7 | 0, 2 | 0, 2 |  |  |  |  |  |  |  |  | 0, 2 | 0, 2 |  | 0 |  |  |
| 89 | Shayganmehr et al ([89](#_ENREF_89)) | 2015 | 6 | Iran | AD | 86 | 4, 32 |  |  |  |  |  | 83 |  |  |  | 4, 32 |  |  |  |  |  |
| 90 | Shoaei et al ([90](#_ENREF_90)) | 2019 | 5 | Iran | ET | 77 | 0, 32 | 0, 2 |  |  |  | 33 |  |  |  |  | 0, 32 | 0, 2 |  | 30 |  |  |
| 91 | Snydman et al ([91](#_ENREF_91)) | 2015 | 4 | USA | AD | 925 | 0, 32 | 166, | 310 |  |  |  | 220 |  |  |  | 33, 2 | 166, 2 | 604 | 310 | 12 |  |
| 92 | Snydman et al ([92](#_ENREF_92)) | 2018 | 4 |  | AD | 89 | 0, 32 | 3, 2 | 15 |  |  | 14 |  |  |  |  | 1, 2 | 3, 2 | 38 | 15 | 2 |  |
| 93 | Spigaglia et al ([93](#_ENREF_93)) | 2008 | 5 |  | ET | 83 |  |  | 0 |  |  |  | 83 |  |  |  |  |  |  |  |  |  |
| 94 | Spigaglia et al ([94](#_ENREF_94)) | 2011 | 4 |  | ET | 316 | 0, 32 | 0, 16 | 316 |  |  |  | 316 |  |  |  | 0, 32 | 0, 16 |  |  |  |  |
| 95 | Spigaglia et al ([95](#_ENREF_95)) | 2017 | 6 | Italy | AD | 50 | 0, 2 | 0, 16 |  |  |  |  |  |  |  |  | 0, 2 | 0, 16 |  |  |  |  |
| 96 | Taori et al ([96](#_ENREF_96)) | 2010 | 3 | UK | AD | 179 | 0, 32 | 0, 32 | 8 |  |  | 171 |  | 12 |  | 24 | 0, 32 | 0, 32 |  |  |  |  |
| 97 | Tenover et al ([97](#_ENREF_97)) | 2012 | 4 | USA | ET | 316 | 0, 32 |  | 120 |  |  | 131 |  |  |  |  | 0, 32 |  |  |  |  |  |
| 98 | Tian et al ([98](#_ENREF_98)) | 2016 | 6 | China | AD | 344 | 0, 32 | 0, 2 |  | 5 |  |  | 338 |  |  |  | 0, 32 | 0, 2 |  |  |  | 0 |
| 99 | Tickler et al ([99](#_ENREF_99)) | 2014 | 4 | USA | AD | 312 | 0, 32 | 37, | 163 |  |  | 126 |  | 11 |  |  | 0, 32 | 37, 2 |  |  |  |  |
| 100 | Tickler et al ([100](#_ENREF_100)) | 2019 | 5 | USA | ET | 940 | 0, 32 | 81, 2 | 269 |  |  | 393 |  | 36 |  |  | 0, 32 | 81, 2 |  |  |  |  |
| 101 | Tkhawkho et al ([101](#_ENREF_101)) | 2017 | 5 | Israel | ET | 81 | 6, 2 | 4, 2 |  |  |  |  |  |  |  |  | 6, 2 | 4, 2 |  | 17 |  |  |
| 102 | Tokimatsu et al ([102](#_ENREF_102)) | 2018 | 5 | Japan | BM | 167 | 0, 32 | 0, 2 |  |  |  |  |  |  |  |  | 0, 32 | 0, 2 |  |  |  |  |
| 103 | Venugopal et al ([103](#_ENREF_103)) | 2012 | 5 | USA | ET | 118 | 0, 32 | 0, |  |  |  |  |  |  |  |  | 0, 32 | 0, 2 |  |  |  |  |
| 104 | Wang et al ([104](#_ENREF_104)) | 2017 | 4 | China | AD | 74 |  | 0, 2 |  |  |  | 27 |  | 1 |  |  |  | 0, 2 |  |  |  |  |
| 105 | Wang et al ([105](#_ENREF_105)) | 2018 | 5 | China | ET | 80 | 0, 32 | 0, 32 |  | 0 | 0 | 77 |  |  |  | 41 | 0, 32 | 0, 32 |  |  |  | 0 |
| 106 | Wieczorkiewicz et al ([106](#_ENREF_106)) | 2015 | 6 | USA | AD | 142 |  |  | 122 |  | 2 | 87 |  |  |  | 142 |  |  |  |  |  |  |
| 107 | Wolfe et al ([107](#_ENREF_107)) | 2018 | 1 | Australia | BM | 100 | 2, 2 | 8, 2 |  |  |  |  |  |  |  |  | 2, 2 | 8, 2 |  |  |  | 0 |
| 108 | Wultańska et al ([108](#_ENREF_108)) | 2010 | 5 | Poland | ET | 40 | 0, 32 | 0, 32 | 0 |  |  |  | 40 |  |  |  | 0, 32 | 0, 32 |  |  |  |  |
| 109 | Yang et al ([109](#_ENREF_109)) | 2017 | 6 | China | AD | 70 | 0, 32 | 0, 4 | 16 |  |  | 37 |  | 13 |  |  | 0, 32 | 0, 4 |  |  |  |  |
| 110 | Zhou et al ([110](#_ENREF_110)) | 2014 | 5 | USA | ET | 46 | 0, 32 |  | 36 |  |  | 4 | 46 | 6 |  |  | 0, 32 |  |  |  |  |  |
| 111 | Zhou et al ([111](#_ENREF_111)) | 2019 | 6 | China | AD | 73 | 0, 32 | 0, 32 | 9 | 0 | 0 | 39 | 52 | 10 |  |  | 0, 32 | 0, 32 |  |  |  |  |

1. Ackermann G, Degner A, Cohen SH, Silva Jr J, Rodloff AC. Prevalence and association of macrolide–lincosamide–streptogramin B (MLSB) resistance with resistance to moxifloxacin in Clostridium difficile. Journal of Antimicrobial Chemotherapy. 2003;51(3):599-603.

2. Ackermann G, Löffler B, Adler D, Rodloff AC. In vitro activity of OPT-80 against Clostridium difficile. Antimicrobial agents and chemotherapy. 2004;48(6):2280-2.

3. Álvarez-Pérez S, Blanco JL, Harmanus C, Kuijper E, García ME. Subtyping and antimicrobial susceptibility of Clostridium difficile PCR ribotype 078/126 isolates of human and animal origin. Veterinary microbiology. 2017;199:15-22.

4. Androga GO, Knight DR, Lim S-C, Foster NF, Riley TV. Antimicrobial resistance in large clostridial toxin-negative, binary toxin-positive Clostridium difficile ribotypes. Anaerobe. 2018;54:55-60.

5. Aoki K, Takeda S, Miki T, Ishii Y, Tateda K. Antimicrobial susceptibility and molecular characterization using whole-genome sequencing of Clostridioides difficile collected in 82 hospitals in Japan between 2014 and 2016. Antimicrobial agents and chemotherapy. 2019;63(12).

6. Aptekorz M, Szczegielniak A, Wiechuła B, Harmanus C, Kuijper E, Martirosian G. Occurrence of Clostridium difficile ribotype 027 in hospitals of Silesia, Poland. Anaerobe. 2017;45:106-13.

7. Arca-Suárez J, Galán-Sánchez F, Cano-Cano F, García-Santos G, Rodríguez-Iglesias M. Antimicrobial susceptibility and molecular typing of toxigenic clinical isolates of Clostridium difficile causing infections in the south of Spain. Anaerobe. 2018;54:146-50.

8. Baghani A, Ghourchian S, Aliramezani A, Yaseri M, Mesdaghinia A, Douraghi M. Highly antibiotic‐resistant Clostridium difficile isolates from Iranian patients. Journal of applied microbiology. 2018;125(5):1518-25.

9. Balassiano IT, dos Santos-Filho J, Vital-Brazil JM, Nouér SA, Souza CR, Brazier JS, et al. Detection of cross-infection associated to a Brazilian PCR-ribotype of Clostridium difficile in a university hospital in Rio de Janeiro, Brazil. Antonie Van Leeuwenhoek. 2011;99(2):249-55.

10. Beran V, Chmelar D, Vobejdova J, Konigova A, Nemec J, Tvrdik J. Sensitivity to antibiotics of Clostridium difficile toxigenic nosocomial strains. Folia microbiologica. 2014;59(3):209-15.

11. Beran V, Kuijper E, Harmanus C, Sanders I, van Dorp S, Knetsch C, et al. Molecular typing and antimicrobial susceptibility testing to six antimicrobials of Clostridium difficile isolates from three Czech hospitals in Eastern Bohemia in 2011–2012. Folia microbiologica. 2017;62(5):445-51.

12. Berger FK, Rasheed SS, Araj GF, Mahfouz R, Rimmani HH, Karaoui WR, et al. Molecular characterization, toxin detection and resistance testing of human clinical Clostridium difficile isolates from Lebanon. International Journal of Medical Microbiology. 2018;308(3):358-63.

13. Bourgault A-M, Lamothe F, Loo VG, Poirier L, group C-Cs. In vitro susceptibility of Clostridium difficile clinical isolates from a multi-institutional outbreak in Southern Quebec, Canada. Antimicrobial agents and chemotherapy. 2006;50(10):3473-5.

14. Büchler AC, Rampini SK, Stelling S, Ledergerber B, Peter S, Schweiger A, et al. Antibiotic susceptibility of Clostridium difficile is similar worldwide over two decades despite widespread use of broad-spectrum antibiotics: an analysis done at the University Hospital of Zurich. BMC infectious diseases. 2014;14(1):607.

15. Byun J-H, Kim H, Kim JL, Kim D, Jeong SH, Shin JH, et al. A nationwide study of molecular epidemiology and antimicrobial susceptibility of Clostridioides difficile in South Korea. Anaerobe. 2019;60:102106.

16. Chatedaki C, Voulgaridi I, Kachrimanidou M, Hrabak J, Papagiannitsis C, Petinaki E. Antimicrobial susceptibility and mechanisms of resistance of Greek Clostridium difficile clinical isolates. Journal of global antimicrobial resistance. 2019;16:53-8.

17. Chen Y-B, Gu S-L, Shen P, Lv T, Fang Y-H, Tang L-L, et al. Molecular epidemiology and antimicrobial susceptibility of Clostridium difficile isolated from hospitals during a 4-year period in China. Journal of medical microbiology. 2018;67(1):52-9.

18. Cheng J-W, Yang Q-W, Xiao M, Yu S-Y, Zhou M-L, Kudinha T, et al. High in vitro activity of fidaxomicin against Clostridium difficile isolates from a university teaching hospital in China. Journal of microbiology, immunology and infection. 2018;51(3):411-6.

19. Chia J-H, Lai H-C, Su L-H, Kuo A-J, Wu T-L. Molecular epidemiology of Clostridium difficile at a medical center in Taiwan: persistence of genetically clustering of A− B+ isolates and increase of A+ B+ isolates. PLoS One. 2013;8(10).

20. Chow VC, Kwong TN, So EW, Ho YI, Wong SH, Lai RW, et al. Surveillance of antibiotic resistance among common Clostridium difficile ribotypes in Hong Kong. Scientific reports. 2017;7(1):1-6.

21. Costa CL, de Carvalho CBM, González RH, Gifoni MAC, de Albuquerque Ribeiro R, Quesada-Gómez C, et al. Molecular epidemiology of Clostridium difficile infection in a Brazilian cancer hospital. Anaerobe. 2017;48:232-6.

22. Dong D, Zhang L, Chen X, Jiang C, Yu B, Wang X, et al. Antimicrobial susceptibility and resistance mechanisms of clinical Clostridium difficile from a Chinese tertiary hospital. International journal of antimicrobial agents. 2013;41(1):80-4.

23. Dong D, Peng Y, Zhang L, Jiang C, Wang X, Mao E. Clinical and microbiological characterization ofClostridium difficileinfection in a tertiary care hospital in Shanghai, China. Chinese medical journal. 2014;127(9):1601-7.

24. Ebrahim-Saraie HS, Heidari H, Amanati A, Bazargani A, Taghavi SA, Nikokar I, et al. A multicenter-based study on epidemiology, antibiotic susceptibility and risk factors of toxigenic Clostridium difficile in hospitalized patients in southwestern Iran. Infez Med. 2018;26(4):308-15.

25. Eckert C, Coignard B, Hebert M, Tarnaud C, Tessier C, Lemire A, et al. Clinical and microbiological features of Clostridium difficile infections in France: the ICD-RAISIN 2009 national survey. Medecine et maladies infectieuses. 2013;43(2):67-74.

26. Eitel Z, Terhes G, Sóki J, Nagy E, Urbán E. Investigation of the MICs of fidaxomicin and other antibiotics against Hungarian Clostridium difficile isolates. Anaerobe. 2015;31:47-9.

27. Fenner L, Frei R, Gregory M, Dangel M, Stranden A, Widmer A. Epidemiology of Clostridium difficile-associated disease at University Hospital Basel including molecular characterisation of the isolates 2006–2007. European journal of clinical microbiology & infectious diseases. 2008;27(12):1201.

28. Fraga EG, Nicodemo AC, Sampaio JLM. Antimicrobial susceptibility of Brazilian Clostridium difficile strains determined by agar dilution and disk diffusion. The Brazilian Journal of Infectious Diseases. 2016;20(5):476-81.

29. Freeman J, Vernon J, Morris K, Nicholson S, Todhunter S, Longshaw C, et al. Pan-European longitudinal surveillance of antibiotic resistance among prevalent Clostridium difficile ribotypes. Clinical Microbiology and Infection. 2015;21(3):248. e9-. e16.

30. Gao Q, Wu S, Huang H, Ni Y, Chen Y, Hu Y, et al. Toxin profiles, PCR ribotypes and resistance patterns of Clostridium difficile: a multicentre study in China, 2012–2013. International journal of antimicrobial agents. 2016;48(6):736-9.

31. Giufrè M, Accogli M, Ricchizzi E, Barbanti F, Farina C, Fazii P, et al. Multidrug-Resistant infections in long-term care facilities: extended-spectrum β-lactamase–producing Enterobacteriaceae and hypervirulent antibiotic resistant Clostridium difficile. Diagnostic microbiology and infectious disease. 2018;91(3):275-81.

32. Goudarzi M, Goudarzi H, Alebouyeh M, Rad MA, Mehr FSS, Zali MR, et al. Antimicrobial susceptibility of Clostridium difficile clinical isolates in Iran. Iranian Red Crescent Medical Journal. 2013;15(8):704.

33. Harvala H, Alm E, Åkerlund T, Rizzardi K. Emergence and spread of moxifloxacin-resistant Clostridium difficile ribotype 231 in Sweden between 2006 and 2015. New microbes and new infections. 2016;14:58-66.

34. Hastey CJ, Dale SE, Nary J, Citron D, Law JH, Roe-Carpenter DE, et al. Comparison of Clostridium difficile minimum inhibitory concentrations obtained using agar dilution vs broth microdilution methods. Anaerobe. 2017;44:73-7.

35. Hecht DW, Galang MA, Sambol SP, Osmolski JR, Johnson S, Gerding DN. In vitro activities of 15 antimicrobial agents against 110 toxigenic Clostridium difficile clinical isolates collected from 1983 to 2004. Antimicrobial agents and chemotherapy. 2007;51(8):2716-9.

36. Hidalgo-Villeda F, Tzoc E, Torres L, Bu E, Rodríguez C, Quesada-Gómez C. Diversity of multidrug-resistant epidemic Clostridium difficile NAP1/RT027/ST01 strains in tertiary hospitals from Honduras. Anaerobe. 2018;52:75-8.

37. Huang H, Wu S, Wang M, Zhang Y, Fang H, Palmgren A-C, et al. Clostridium difficile infections in a Shanghai hospital: antimicrobial resistance, toxin profiles and ribotypes. International journal of antimicrobial agents. 2009;33(4):339-42.

38. Huang H, Weintraub A, Fang H, Wu S, Zhang Y, Nord CE. Antimicrobial susceptibility and heteroresistance in Chinese Clostridium difficile strains. Anaerobe. 2010;16(6):633-5.

39. Hung Y-P, Tsai P-J, Lee Y-T, Tang H-J, Lin H-J, Liu H-C, et al. Nationwide surveillance of ribotypes and antimicrobial susceptibilities of toxigenic Clostridium difficile isolates with an emphasis on reduced doxycycline and tigecycline susceptibilities among ribotype 078 lineage isolates in Taiwan. Infection and drug resistance. 2018;11:1197.

40. Jamal WY, Mokaddas EM, Verghese TL, Rotimi V. In vitro activity of 15 antimicrobial agents against clinical isolates of Clostridium difficile in Kuwait. International journal of antimicrobial agents. 2002;20(4):270-4.

41. Jamal WY, Rotimi VO. Surveillance of antibiotic resistance among hospital-and community-acquired toxigenic Clostridium difficile isolates over 5-year period in Kuwait. PloS one. 2016;11(8).

42. Jiang Z, DuPont H, La Rocco M, Garey K. In vitro susceptibility of Clostridium difficile to rifaximin and rifampin in 359 consecutive isolates at a university hospital in Houston, Texas. Journal of clinical pathology. 2010;63(4):355-8.

43. Jiménez A, Araya R, Paniagua D, Camacho-Mora Z, Du T, Golding G, et al. Molecular epidemiology and antimicrobial resistance of Clostridium difficile in a national geriatric hospital in Costa Rica. Journal of Hospital Infection. 2018;99(4):475-80.

44. Jin D, Luo Y, Huang C, Cai J, Ye J, Zheng Y, et al. Molecular epidemiology of Clostridium difficile infection in hospitalized patients in eastern China. Journal of clinical microbiology. 2017;55(3):801-10.

45. John R, Brazier J. Antimicrobial susceptibility of polymerase chain reaction ribotypes of Clostridium difficile commonly isolated from symptomatic hospital patients in the UK. Journal of Hospital Infection. 2005;61(1):11-4.

46. Karlowsky JA, Zhanel GG, Hammond GW, Rubinstein E, Wylie J, Du T, et al. Multidrug-resistant North American pulsotype 2 Clostridium difficile was the predominant toxigenic hospital-acquired strain in the province of Manitoba, Canada, in 2006–2007. Journal of medical microbiology. 2012;61(5):693-700.

47. Karlowsky JA, Adam HJ, Kosowan T, Baxter MR, Nichol KA, Laing NM, et al. PCR ribotyping and antimicrobial susceptibility testing of isolates of Clostridium difficile cultured from toxin-positive diarrheal stools of patients receiving medical care in Canadian hospitals: the Canadian Clostridium difficile Surveillance Study (CAN-DIFF) 2013–2015. Diagnostic microbiology and infectious disease. 2018;91(2):105-11.

48. Kim J, Kang JO, Pai H, Choi TY. Association between PCR ribotypes and antimicrobial susceptibility among Clostridium difficile isolates from healthcare-associated infections in South Korea. International journal of antimicrobial agents. 2012;40(1):24-9.

49. Knight DR, Giglio S, Huntington PG, Korman TM, Kotsanas D, Moore CV, et al. Surveillance for antimicrobial resistance in Australian isolates of Clostridium difficile, 2013–14. Journal of Antimicrobial Chemotherapy. 2015;70(11):2992-9.

50. Knight DR, Riley TV. Clostridium difficile clade 5 in Australia: antimicrobial susceptibility profiling of PCR ribotypes of human and animal origin. Journal of Antimicrobial Chemotherapy. 2016;71(8):2213-7.

51. Kociolek LK, Gerding DN, Osmolski JR, Patel SJ, Snydman DR, McDermott LA, et al. Differences in the molecular epidemiology and antibiotic susceptibility of Clostridium difficile isolates in pediatric and adult patients. Antimicrobial agents and chemotherapy. 2016;60(8):4896-900.

52. Kouhsari E, Douraghi M, Krutova M, Yaseri HF, Talebi M, Baseri Z, et al. The emergence of metronidazole and vancomycin reduced susceptibility in Clostridium difficile isolates in Iran. Journal of global antimicrobial resistance. 2019;18:28-33.

53. Krutova M, Matejkova J, Tkadlec J, Nyc O. Antibiotic profiling of Clostridium difficile ribotype 176–A multidrug resistant relative to C. difficile ribotype 027. Anaerobe. 2015;36:88-90.

54. Kullin BR, Reid S, Abratt V. Clostridium difficile in patients attending tuberculosis hospitals in Cape Town, South Africa, 2014-2015. African journal of laboratory medicine. 2018;7(2):1-9.

55. Kuwata Y, Tanimoto S, Sawabe E, Shima M, Takahashi Y, Ushizawa H, et al. Molecular epidemiology and antimicrobial susceptibility of Clostridium difficile isolated from a university teaching hospital in Japan. European Journal of Clinical Microbiology & Infectious Diseases. 2015;34(4):763-72.

56. Lachowicz D, Pituch H, Obuch-Woszczatyński P. Antimicrobial susceptibility patterns of Clostridium difficile strains belonging to different polymerase chain reaction ribotypes isolated in Poland in 2012. Anaerobe. 2015;31:37-41.

57. Li H, Li W-G, Zhang W-Z, Yu S-B, Liu Z-J, Zhang X, et al. Antibiotic resistance of clinical isolates of Clostridioides difficile in China and its association with geographical regions and patient age. Anaerobe. 2019;60:102094.

58. Liao C-H, Ko W-C, Lu J-J, Hsueh P-R. Characterizations of clinical isolates of Clostridium difficile by toxin genotypes and by susceptibility to 12 antimicrobial agents, including fidaxomicin (OPT-80) and rifaximin: a multicenter study in Taiwan. Antimicrobial agents and chemotherapy. 2012;56(7):3943-9.

59. Lidan C, Linhai L, Yang L, Zhaohui S, Xiaoyan H, Yuling S. Molecular characterization and antimicrobial susceptibility of tcdA-negative Clostridium difficile isolates from Guangzhou, China. Diagnostic microbiology and infectious disease. 2016;84(4):361-5.

60. López-Ureña D, Quesada-Gómez C, Miranda E, Fonseca M, Rodríguez-Cavallini E. Spread of epidemic Clostridium difficile NAP1/027 in Latin America: case reports in Panama. Journal of medical microbiology. 2014;63(2):322-4.

61. López-Ureña D, Quesada-Gómez C, Montoya-Ramírez M, del Mar Gamboa-Coronado M, Somogyi T, Rodríguez C, et al. Predominance and high antibiotic resistance of the emerging Clostridium difficile genotypes NAPCR1 and NAP9 in a Costa Rican hospital over a 2-year period without outbreaks. Emerging microbes & infections. 2016;5(1):1-5.

62. Luo Y, Zhang W, Cheng J-W, Xiao M, Sun G-R, Guo C-J, et al. Molecular epidemiology of Clostridium difficile in two tertiary care hospitals in Shandong Province, China. Infection and drug resistance. 2018;11:489.

63. Mutlu E, Wroe AJ, Sanchez-Hurtado K, Brazier JS, Poxton IR. Molecular characterization and antimicrobial susceptibility patterns of Clostridium difficile strains isolated from hospitals in south-east Scotland. Journal of medical microbiology. 2007;56(7):921-9.

64. Nasereddin LM, Bakri FG, Shehabi AA. Clostridium difficile infections among Jordanian adult hospitalized patients. American journal of infection control. 2009;37(10):864-6.

65. Ngamskulrungroj P, Sanmee S, Pusathit P, Piewngam P, Elliott B, Riley TV, et al. Molecular epidemiology of Clostridium difficile infection in a large teaching hospital in Thailand. PloS one. 2015;10(5).

66. Niyogi SK. Antimicrobial susceptibility of Clostridium difficile strains isolated from hospitalised patients with acute diarrhoea. Journal of diarrhoeal diseases research. 1992:156-8.

67. Novak A, Spigaglia P, Barbanti F, Goic-Barisic I, Tonkic M. First clinical and microbiological characterization of Clostridium difficile infection in a Croatian University Hospital. Anaerobe. 2014;30:18-23.

68. Obuch-Woszczatyński P, Dubiel G, Harmanus C, Kuijper E, Duda U, Wultańska D, et al. Emergence of Clostridium difficile infection in tuberculosis patients due to a highly rifampicin-resistant PCR ribotype 046 clone in Poland. European journal of clinical microbiology & infectious diseases. 2013;32(8):1027-30.

69. Oka K, Osaki T, Hanawa T, Kurata S, Okazaki M, Manzoku T, et al. Molecular and microbiological characterization of Clostridium difficile isolates from single, relapse, and reinfection cases. Journal of clinical microbiology. 2012;50(3):915-21.

70. Pelaez T, Alcala L, Alonso R, Rodriguez-Creixems M, Garcia-Lechuz J, Bouza E. Reassessment of Clostridium difficile susceptibility to metronidazole and vancomycin. Antimicrobial agents and chemotherapy. 2002;46(6):1647-50.

71. Peng Z, Addisu A, Alrabaa S, Sun X. Antibiotic resistance and toxin production of Clostridium difficile isolates from the hospitalized patients in a large hospital in Florida. Frontiers in microbiology. 2017;8:2584.

72. Peretz A, Tkhawkho L, Pastukh N, Brodsky D, Halevi CN, Nitzan O. Correlation between fecal calprotectin levels, disease severity and the hypervirulent ribotype 027 strain in patients with Clostridium difficile infection. BMC infectious diseases. 2016;16(1):309.

73. Piepenbrock E, Stelzer Y, Berger F, Jazmati N. Changes in Clostridium (Clostridioides) difficile PCR-Ribotype Distribution and Antimicrobial Resistance in a German Tertiary Care Hospital Over the Last 10 Years. Current microbiology. 2019;76(4):520-6.

74. Pinto LJ, Alcides AP, Ferreira EO, Avelar KE, Sabrá A, Domingues RM, et al. Incidence and importance of Clostridium difficile in paediatric diarrhoea in Brazil. Journal of medical microbiology. 2003;52(12):1095-9.

75. Pirš T, Avberšek J, Zdovc I, Krt B, Andlovic A, Lejko-Zupanc T, et al. Antimicrobial susceptibility of animal and human isolates of Clostridium difficile by broth microdilution. Journal of medical microbiology. 2013;62(9):1478-85.

76. Putsathit P, Maneerattanaporn M, Piewngam P, Knight DR, Kiratisin P, Riley TV. Antimicrobial susceptibility of Clostridium difficile isolated in Thailand. Antimicrobial Resistance & Infection Control. 2017;6(1):58.

77. Ramírez-Vargas G, Quesada-Gómez C, Acuña-Amador L, López-Ureña D, Murillo T, del Mar Gamboa-Coronado M, et al. A Clostridium difficile lineage endemic to Costa Rican hospitals is multidrug resistant by acquisition of chromosomal mutations and novel mobile genetic elements. Antimicrobial agents and chemotherapy. 2017;61(4):e02054-16.

78. Reil M, Hensgens M, Kuijper E, Jakobiak T, Gruber H, Kist M, et al. Seasonality of Clostridium difficile infections in Southern Germany. Epidemiology & Infection. 2012;140(10):1787-93.

79. Roberts S, Heffernan H, Al Anbuky N, Pope C, Paviour S, Camp T, et al. Molecular epidemiology and susceptibility profiles of Clostridium difficile in New Zealand, 2009. NZ Med J. 2011;124(1332):45-51.

80. Rodriguez C, Avesani V, Taminiau B, Van Broeck J, Brévers B, Delmée M, et al. Investigation of Clostridium difficile interspecies relatedness using multilocus sequence typing, multilocus variable-number tandem-repeat analysis and antimicrobial susceptibility testing. The Veterinary Journal. 2015;206(3):349-55.

81. Russello G, Russo A, Sisto F, Scaltrito MM, Farina C. Laboratory diagnosis of Clostridium difficile associated diarrhoea and molecular characterization of clinical isolates. New Microbiologica. 2012;35(3):307-16.

82. Saatian B, Banerjee C, Carroll KC, Ross TL, Kamangar F. Characterization of clostridium difficile infection and analysis of recovered isolates in a community hospital population in Baltimore, Maryland. Infectious Diseases in Clinical Practice. 2010;18(6):383-8.

83. Samonis G, Vardakas K, Tansarli G, Dimopoulou D, Papadimitriou G, Kofteridis D, et al. Clostridium difficile in Crete, Greece: epidemiology, microbiology and clinical disease. Epidemiology & Infection. 2016;144(1):161-70.

84. Sandell S, Rashid M-U, Jorup-Rönström C, Ellström K, Nord CE, Weintraub A. Clostridium difficile recurrences in Stockholm. Anaerobe. 2016;38:97-102.

85. Santos A, Isidro J, Silva C, Boaventura L, Diogo J, Faustino A, et al. Molecular and epidemiologic study of Clostridium difficile reveals unusual heterogeneity in clinical strains circulating in different regions in Portugal. Clinical Microbiology and Infection. 2016;22(8):695-700.

86. Secco DA, Balassiano IT, Boente RF, Miranda KR, Brazier J, Hall V, et al. Clostridium difficile infection among immunocompromised patients in Rio de Janeiro, Brazil and detection of moxifloxacin resistance in a ribotype 014 strain. Anaerobe. 2014;28:85-9.

87. Seo M-R, Kim J, Lee Y, Lim D-G, Pai H. Prevalence, genetic relatedness and antibiotic resistance of hospital-acquired Clostridium difficile PCR ribotype 018 strains. International journal of antimicrobial agents. 2018;51(5):762-7.

88. Seugendo M, Mshana S, Hokororo A, Okamo B, Mirambo M, von Müller L, et al. Clostridium difficile infections among adults and children in Mwanza/Tanzania: is it an underappreciated pathogen among immunocompromised patients in sub-Saharan Africa? New microbes and new infections. 2015;8:99-102.

89. Shayganmehr F-S, Alebouyeh M, Azimirad M, Aslani MM, Zali MR. Association of tcdA+/tcdB+ Clostridium difficile genotype with emergence of multidrug-resistant strains conferring metronidazole resistant phenotype. Iranian biomedical journal. 2015;19(3):143.

90. Shoaei P, Shojaei H, Khorvash F, Hosseini SM, Ataei B, Tavakoli H, et al. Molecular epidemiology of Clostridium difficile infection in Iranian hospitals. Antimicrobial Resistance & Infection Control. 2019;8(1):12.

91. Snydman D, McDermott L, Jacobus N, Thorpe C, Stone S, Jenkins S, et al. US-based national sentinel surveillance study for the epidemiology of Clostridium difficile-associated diarrheal isolates and their susceptibility to fidaxomicin. Antimicrobial agents and chemotherapy. 2015;59(10):6437-43.

92. Snydman DR, McDermott LA, Thorpe CM, Chang J, Wick J, Walk ST, et al. Antimicrobial susceptibility and ribotypes of Clostridium difficile isolates from a Phase 2 clinical trial of ridinilazole (SMT19969) and vancomycin. Journal of Antimicrobial Chemotherapy. 2018;73(8):2078-84.

93. Spigaglia P, Barbanti F, Mastrantonio P, Brazier JS, Barbut F, Delmée M, et al. Fluoroquinolone resistance in Clostridium difficile isolates from a prospective study of C. difficile infections in Europe. Journal of medical microbiology. 2008;57(6):784-9.

94. Spigaglia P, Barbanti F, Mastrantonio P, difficile ESGoC, Ackermann G, Balmelli C, et al. Multidrug resistance in European Clostridium difficile clinical isolates. Journal of antimicrobial chemotherapy. 2011;66(10):2227-34.

95. Spigaglia P, Barbanti F, Castagnola E, Diana MC, Pescetto L, Bandettini R. Clostridium difficile causing pediatric infections: new findings from a hospital-based study in Italy. Anaerobe. 2017;48:262-8.

96. Taori SK, Hall V, Poxton IR. Changes in antibiotic susceptibility and ribotypes in Clostridium difficile isolates from southern Scotland, 1979–2004. Journal of medical microbiology. 2010;59(3):338-44.

97. Tenover FC, Tickler IA, Persing DH. Antimicrobial-resistant strains of Clostridium difficile from North America. Antimicrobial agents and chemotherapy. 2012;56(6):2929-32.

98. Tian T-t, Zhao J-h, Yang J, Qiang C-x, Li Z-r, Chen J, et al. Molecular characterization of Clostridium difficile isolates from human subjects and the environment. PloS one. 2016;11(3).

99. Tickler IA, Goering RV, Whitmore JD, Lynn AN, Persing DH, Tenover FC. Strain types and antimicrobial resistance patterns of Clostridium difficile isolates from the United States, 2011 to 2013. Antimicrobial agents and chemotherapy. 2014;58(7):4214-8.

100. Tickler IA, Obradovich AE, Goering RV, Fang FC, Tenover FC, Consortium H. Changes in molecular epidemiology and antimicrobial resistance profiles of Clostridioides (Clostridium) difficile strains in the United States between 2011 and 2017. Anaerobe. 2019;60:102050.

101. Tkhawkho L, Nitzan O, Pastukh N, Brodsky D, Jackson K, Peretz A. Antimicrobial susceptibility of Clostridium difficile isolates in Israel. Journal of global antimicrobial resistance. 2017;10:161-4.

102. Tokimatsu I, Shigemura K, Osawa K, Kinugawa S, Kitagawa K, Nakanishi N, et al. Molecular epidemiologic study of Clostridium difficile infections in university hospitals: Results of a nationwide study in Japan. Journal of infection and chemotherapy. 2018;24(8):641-7.

103. Venugopal AA, Riederer K, Patel SM, Szpunar S, Jahamy H, Valenti S, et al. Lack of association of outcomes with treatment duration and microbiologic susceptibility data in Clostridium difficile infections in a non-NAP1/BI/027 setting. Scandinavian journal of infectious diseases. 2012;44(4):243-9.

104. Wang R, Suo L, Chen HX, Song LJ, Shen YY, Luo YP. Molecular epidemiology and antimicrobial susceptibility of Clostridium difficile isolated from the Chinese People’s Liberation Army General Hospital in China. International Journal of Infectious Diseases. 2018;67:86-91.

105. Wang B, Lv Z, Zhang P, Su J. Molecular epidemiology and antimicrobial susceptibility of human Clostridium difficile isolates from a single institution in Northern China. Medicine. 2018;97(25).

106. Wieczorkiewicz JT, Lopansri BK, Cheknis A, Osmolski JR, Hecht DW, Gerding DN, et al. Fluoroquinolone and macrolide exposure predict Clostridium difficile infection with the highly fluoroquinolone-and macrolide-resistant epidemic C. difficile strain BI/NAP1/027. Antimicrobial agents and chemotherapy. 2016;60(1):418-23.

107. Wolfe C, Pagano P, Pillar CM, Shinabarger DL, Boulos RA. Comparison of the in vitro antibacterial activity of Ramizol, fidaxomicin, vancomycin, and metronidazole against 100 clinical isolates of Clostridium difficile by broth microdilution. Diagnostic microbiology and infectious disease. 2018;92(3):250-2.

108. Wultańska D, Banaszkiewicz A, Radzikowski A, Obuch-Woszczatyński P, Młynarczyk G, Brazier J, et al. Clostridium difficile infection in Polish pediatric outpatients with inflammatory bowel disease. European journal of clinical microbiology & infectious diseases. 2010;29(10):1265-70.

109. Yang J, Zhang X, Liu X, Cai L, Feng P, Wang X, et al. Antimicrobial susceptibility of Clostridium difficile isolates from ICU colonized patients revealed alert to ST-37 (RT 017) isolates. Diagnostic microbiology and infectious disease. 2017;89(2):161-3.

110. Zhou Y, Burnham C-AD, Hink T, Chen L, Shaikh N, Wollam A, et al. Phenotypic and genotypic analysis of Clostridium difficile isolates: a single-center study. Journal of clinical microbiology. 2014;52(12):4260-6.

111. Zhou Y, Mao L, Yu J, Lin Q, Luo Y, Zhu X, et al. Epidemiology of Clostridium difficile infection in hospitalized adults and the first isolation of C. difficile PCR ribotype 027 in central China. BMC infectious diseases. 2019;19(1):232.
