# Supplementary material for: Antimicrobial resistance in Clostridioides (Clostridium) difficile derived from humans: a systematic review and meta-analysis
Source: Antimicrob Resist Infect Control. 2020 Sep 25;9:158. doi: 10.1186/s13756-020-00815-5 (PMC7517813; doi:10.1186/s13756-020-00815-5)
Supplement: Supplementary file 2 — Additional file 2. [file 13756_2020_815_MOESM2_ESM.docx]

| **First Author, Year of Publication** | **Selection (3 points)** | **Comparability (2 points)** | **Outcome (3 points)** | **Total (8 points)** |
| --- | --- | --- | --- | --- |
| Ackermann et al, 2003 | 2 | 1 | 1 | 4 |
| Ackermann et al, 2004 | 2 | 0 | 2 | 4 |
| Álvarez-Pérez et al, 2017 | 1 | 0 | 1 | 2 |
| Androga et al, 2018 | 1 | 1 | 2 | 4 |
| Aoki et al, 2019 | 2 | 0 | 3 | 5 |
| Aptekorz et al, 2017 | 2 | 1 | 1 | 3 |
| Arca-Suárez et al, 2018 | 2 | 1 | 1 | 4 |
| Baghani et al, 2018 | 2 | 1 | 2 | 5 |
| Balassiano et al, 2011 | 2 | 1 | 1 | 4 |
| Beran et al, 2014 | 2 | 1 | 3 | 6 |
| Beran et al, 2017 | 2 | 1 | 3 | 6 |
| Berger et al, 2018 | 2 | 1 | 1 | 4 |
| Bourgault et al, 2006 | 1 | 1 | 2 | 4 |
| Büchler et al, 2014 | 2 | 1 | 2 | 5 |
| Byun et al, 2019 | 2 | 1 | 3 | 6 |
| Chatedaki et al, 2019 | 1 | 1 | 2 | 4 |
| Chen et al, 2018 | 2 | 1 | 1 | 4 |
| Cheng et al, 2017 | 2 | 1 | 3 | 6 |
| Chia et al, 2013 | 1 | 1 | 2 | 4 |
| Chow et al, 2017 | 1 | 0 | 2 | 3 |
| Costa et al, 2017 | 2 | 1 | 2 | 5 |
| Dong et al, 2013 | 1 | 1 | 2 | 4 |
| Dong et al, 2014 | 2 | 1 | 3 | 6 |
| Ebrahim-Saraie et al, 2018 | 2 | 1 | 2 | 5 |
| Eckert et al, 2013 | 2 | 1 | 1 | 4 |
| Eitel et al, 2014 | 2 | 0 | 1 | 3 |
| Fenner et al, 2008 | 2 | 1 | 2 | 5 |
| Fraga et al, 2016 | 2 | 1 | 2 | 5 |
| Freeman et al, 2014 | 2 | 1 | 2 | 5 |
| Gao et al, 2016 | 2 | 1 | 2 | 5 |
| Giufrè et al, 2018 | 1 | 0 | 2 | 3 |
| Goudarzi et al, 2013 | 2 | 1 | 2 | 5 |
| Harvala et al, 2016 | 1 | 0 | 2 | 3 |
| Hastey et al, 2017 | 1 | 0 | 2 | 3 |
| Hecht et al, 2007 | 2 | 0 | 2 | 4 |
| hidalgo-Villeda et al, 2018 | 2 | 1 | 1 | 4 |
| Huang et al, 2009 | 2 | 1 | 3 | 6 |
| Huang et al, 2010 | 2 | 1 | 2 | 5 |
| Hung et al, 2018 | 1 | 1 | 3 | 5 |
| Jamal et al, 2002 | 2 | 1 | 2 | 5 |
| Jamal et al, 2016 | 2 | 1 | 2 | 5 |
| Jiang et al, 2010 | 2 | 1 | 1 | 4 |
| Jiménez et al, 2018 | 2 | 1 | 1 | 4 |
| Jin et al, 2017 | 2 | 1 | 2 | 5 |
| John et al, 2005 | 2 | 1 | 2 | 5 |
| Karlowsky et al, 2012 | 1 | 1 | 2 | 4 |
| Karlowsky et al, 2018 | 2 | 1 | 3 | 6 |
| Kim et al, 2012 | 2 | 1 | 2 | 5 |
| Knight et al, 2015 | 3 | 1 | 2 | 6 |
| Knight et al, 2016 | 1 | 1 | 3 | 5 |
| Kociolek et al, 2016 | 2 | 1 | 3 | 6 |
| Kouhsari et al, 2019 | 2 | 1 | 2 | 5 |
| Krutova et al, 2015 | 2 | 1 | 3 | 2 |
| Kullin et al, 2018 | 2 | 0 | 1 | 3 |
| Kuwata et al, 2014 | 1 | 0 | 1 | 6 |
| Lachowicz et al, 2014 | 3 | 1 | 1 | 5 |
| Li et al, 2019 | 2 | 1 | 2 | 5 |
| Liao et al, 2012 | 2 | 1 | 2 | 5 |
| Lidan et al, 2016 | 2 | 1 | 1 | 4 |
| Lopez-Urena et al, 2014 | 1 | 1 | 1 | 3 |
| López-Ureña et al, 2016 | 2 | 1 | 1 | 4 |
| Luo et al, 2018 | 2 | 1 | 3 | 6 |
| Mutlu et al, 2007 | 2 | 1 | 2 | 5 |
| Nasereddin et al, 2009 | 2 | 1 | 2 | 5 |
| Ngamskulrungroj et al, 2015 | 2 | 1 | 2 | 5 |
| Niyogi et al, 1992 | 1 | 1 | 2 | 4 |
| Novak et al, 2014 | 2 | 1 | 1 | 4 |
| Obuch-Woszczatyński et al, 2013 | 1 | 0 | 2 | 3 |
| Oka et al, 2011 | 1 | 1 | 2 | 4 |
| Peláez et al, 2002 | 2 | 1 | 3 | 6 |
| Peng et al, 2017 | 2 | 1 | 2 | 5 |
| Peretz et al, 2016 | 2 | 1 | 2 | 5 |
| Piepenbrock et al, 2019 | 2 | 1 | 2 | 5 |
| Pinto et al, 2003 | 2 | 1 | 3 | 6 |
| Pirs et al, 2013 | 1 | 1 | 1 | 3 |
| Putsathit et al, 2017 | 1 | 1 | 2 | 4 |
| Ramírez-Vargas et al, 2017 | 1 | 1 | 1 | 3 |
| Reil et al, 2012 | 2 | 1 | 2 | 5 |
| Roberts et al, 2011 | 1 | 1 | 2 | 4 |
| Rodriguez et al, 2015 | 1 | 1 | 1 | 3 |
| Russello et al, 2012 | 2 | 1 | 1 | 4 |
| Saatian et al, 2010 | 2 | 1 | 2 | 5 |
| Samonis et al, 2016 | 2 | 1 | 2 | 5 |
| Sandell et al, 2016 | 1 | 0 | 3 | 4 |
| Santos et al, 2016 | 3 | 1 | 2 | 6 |
| Secco et al, 2014 | 2 | 1 | 1 | 4 |
| Seo et al, 2018 | 2 | 1 | 2 | 5 |
| Seugendo et al, 2015 | 2 | 1 | 3 | 4 |
| Shayganmehr et al, 2015 | 2 | 1 | 3 | 6 |
| Shoaei et al, 2019 | 2 | 1 | 2 | 5 |
| Snydman et al, 2015 | 1 | 1 | 2 | 4 |
| Snydman et al, 2018 | 1 | 0 | 3 | 4 |
| Spigaglia et al, 2008 | 2 | 1 | 2 | 5 |
| Spigaglia et al, 2011 | 2 | 1 | 1 | 4 |
| Spigaglia et al, 2017 | 2 | 1 | 3 | 6 |
| Taori et al, 2010 | 1 | 0 | 2 | 3 |
| Tenover et al, 2012 | 2 | 1 | 1 | 4 |
| Tian et al, 2016 | 2 | 1 | 3 | 6 |
| Tickler et al, 2014 | 1 | 1 | 2 | 4 |
| Tickler et al, 2019 | 2 | 1 | 2 | 5 |
| Tkhawkho et al, 2017 | 2 | 1 | 2 | 5 |
| Tokimatsu et al, 2018 | 2 | 1 | 2 | 5 |
| Venugopal et al, 2012 | 2 | 1 | 2 | 5 |
| Wang et al, 2017 | 2 | 1 | 1 | 5 |
| Wang et al, 2018 | 2 | 1 | 2 | 4 |
| Wieczorkiewicz et al, 2015 | 2 | 1 | 3 | 6 |
| Wolfe et al, 2018 | 0 | 0 | 1 | 1 |
| Wultańska et al, 2010 | 2 | 1 | 2 | 5 |
| Yang et al, 2017 | 2 | 1 | 3 | 6 |
| Zhou et al, 2014 | 2 | 1 | 2 | 5 |
| Zhou et al, 2019 | 2 | 1 | 3 | 6 |
