# Supplementary figures and images for: Antimicrobial resistance in Clostridioides (Clostridium) difficile derived from humans: a systematic review and meta-analysis
Source: Antimicrob Resist Infect Control. 2020 Sep 25;9:158. doi: 10.1186/s13756-020-00815-5 (PMC7517813; doi:10.1186/s13756-020-00815-5)

**CLSI**


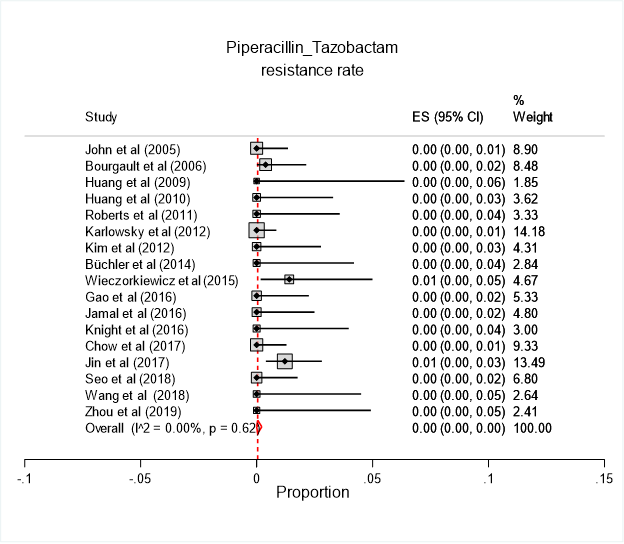


**EUCAST**

Supplement: Supplementary file 3 — Additional file 3. [file 13756_2020_815_MOESM3_ESM.docx]
