# Supplementary material for: Antimicrobial resistance in Clostridioides (Clostridium) difficile derived from humans: a systematic review and meta-analysis
Source: Antimicrob Resist Infect Control. 2020 Sep 25;9:158. doi: 10.1186/s13756-020-00815-5 (PMC7517813; doi:10.1186/s13756-020-00815-5)
Supplement: Supplementary file 4 — Additional file 4. [file 13756_2020_815_MOESM4_ESM.docx]

| Number of isolates | Number of Resistance isolate | % weight | Heterogeneity between groups (P-Value) | Heterogeneity | Poled resistance rate | Metronidazole (breakpoint 2) |
| --- | --- | --- | --- | --- | --- | --- |
| 5900 | 190 | 100 | 0.00 | 91.97 | 0.01 (0.00-0.03) | Overall |
| Year Subgroups analysis | | | | | | |
| 347 | 2 | 13.03 | 0.058 | 0.00 | 0.00  (0.00-0.01) | 1992-2014 |
| 5553 | 188 | 86.97 |  | 92.77 | 0.02  (0.00-0.04) | 2015-2019 |
| Continent Subgroups analysis | | | | | | |
| 1356 | 29 | 43.00 | 0.712 | 80.47 | 0.01  (0.00-0.02) | Europe |
| 3244 | 117 | 14.61 |  | 97.82 | 0.03  (0.00-0.08) | North America |
| 1024 | 42 | 28.98 |  | 95.21 | 0.04  (0.00-0.12) | Asia |
| 219 | 2 | 9.16 |  | - | 0.00  (0.00-0.02) | Oceania |
| 50 | 0 | 2.96 |  | - | 0.00  (0.00-0.07) | South America |
| 7 | 0 | 1.29 |  | - | 0.00  (0.00-0.41) | Africa |
| Quality Subgroups analysis | | | | | | |
| 120 | 2 | 5.56 | 0.998 | - | 0.01  (0.00-0.04) | Low quality |
| 2663 | 128 | 42.58 |  | 89.70 | 0.01  (0.00-0.04) | Moderate quality |
| 3117 | 60 | 51.85 |  | 92.34 | 0.02  (0.00-0.04) | High quality |
| Method Subgroups analysis | | | | | | |
| 4497 | 132 | 52.41 | 0.523 | 93.37 | 0.01  (0.00-0.03) | Agar dilution |
| 1211 | 56 | 40.95 |  | 91.55 | 0.02  (0.00-0.07) | E-test |
| 192 | 2 | 6.64 |  | - | 0.01  (0.00-0.03) | Broth dilution |

| Number of isolates | Number of Resistance isolate | % weight | Heterogeneity between groups (P-Value) | Heterogeneity | Poled resistance rate | Metronidazole (breakpoint 32) |
| --- | --- | --- | --- | --- | --- | --- |
| 13207 | 129 | 100 | 0.00 | 81.40 | 0.00  (0.00,0.00) | Overall |
| Year Subgroups analysis | | | | | | |
| 4759 | 30 | 47.64 | 0.280 | 62.07 | 0.00  (0.00,0.00) | 1992-2014 |
| 8448 | 99 | 52.36 |  | 87.73 | 0.00  (0.00,0.01) | 2015-2019 |
| Continent Subgroups analysis | | | | | | |
| 2122 | 37 | 20.25 | 0.038 | 81.68 | 0.01  (0.00,0.02) | Europe |
| 4928 | 1 | 26.16 |  | 0.00 | 0.00  (0.00,0.00) | North America |
| 5460 | 91 | 46.70 |  | 86.97 | 0.00  (0.00,0.01) | Asia |
| 541 | 0 | 3.49 |  | - | 0.00  (0.00,0.00) | Oceania |
| 73 | 0 | 1.72 |  | - | 0.00  (0.00,0.00) | South America |
| 77 | 0 | 1.39 |  | - | 0.00  (0.00,0.05) | Africa |
| 6 | 0 | 0.28 |  | - | 0.00 (0.00,0.46) | Central America |
| Quality Subgroups analysis | | | | | | |
| 50 | 2 | 1.17 | 0.001 | - | 0.04  (0.00,0.14) | Low quality |
| 5778 | 2 | 41.01 |  | 0.00 | 0.00  (0.00,0.00) | Moderate quality |
| 7379 | 125 | 57.82 |  | 87.91 | 0.00  (0.00,0.01) | High quality |
| Method Subgroups analysis | | | | | | |
| 7933 | 49 | 52.10 | 0.281 | 73.83 | 0.00  (0.00,0.00) | Agar dilution |
| 4968 | 80 | 44.53 |  | 85.86 | 0.00  (0.00,0.00) | E-test |
| 306 | 0 | 3.38 |  | - | 0.00  (0.00,0.01) | Broth dilution |

| Number of isolates | Number of Resistance isolate | % weight | Heterogeneity between groups (P-Value) | Heterogeneity | Poled resistance rate | Vancomycin (breakpoint 32) |
| --- | --- | --- | --- | --- | --- | --- |
| 2307 | 13 | 100 | 0.05 | 38.60 | 0.00  (0.00,0.00) | Overall |

| Number of isolates | Number of Resistance isolate | % weight | Heterogeneity between groups (P-Value) | Heterogeneity | Poled resistance rate | Vancomycin (breakpoint 16) |
| --- | --- | --- | --- | --- | --- | --- |
| 2296 | 10 | 100 | 0.37 | 7.62 | 0.00  (0.00,0.00) | Overall |

| Number of isolates | Number of Resistance isolate | % weight | Heterogeneity between groups (P-Value) | Heterogeneity | Poled resistance rate | Vancomycin (breakpoint 4) |
| --- | --- | --- | --- | --- | --- | --- |
| 1107 | 7 | 100 | 0.05 | 50.10 | 0.00  (0.00,0.01) | Overall |

| Number of isolates | Number of Resistance isolate | % weight | Heterogeneity between groups (P-Value) | Heterogeneity | Poled resistance rate | Vancomycin (breakpoint 2) |
| --- | --- | --- | --- | --- | --- | --- |
| 11188 | 416 | 100 | 0.00 | 93.89 | 0.01  (0.00,0.02) | Overall |
| Year Subgroups analysis | | | | | | |
| 2061 | 61 | 23.14 | 0.48 | 87.48 | 0.01  (0.00,0.03) | 1992-2014 |
| 9127 | 355 | 76.86 |  | 94.74 | 0.01  (0.00,0.03) | 2015-2019 |
| Continent Subgroups analysis | | | | | | |
| 2060 | 20 | 33.52 | 0.000 | 75.26 | 0.00  (0.00,0.01) | Europe |
| 5087 | 332 | 17.34 |  | 98.14 | 0.04  (0.01,0.09) | North America |
| 3321 | 27 | 39.07 |  | 67.00 | 0.00  (0.00,0.01) | Asia |
| 659 | 8 | 7.08 |  | 87.57 | 0.01  (0.00,0.05) | Oceania |
| 54 | 29 | 2.21 |  | - | 0.53  (0.38,0.68) | South America |
| 7 | 0 | 0.78 |  | - | 0.00  (0.00,0.41) | Africa |
| Quality Subgroups analysis | | | | | | |
| 120 | 8 | 3.12 | 0.01 | - | 0.06  (0.02,0.11) | Low quality |
| 4460 | 276 | 39.86 |  | 94.53 | 0.02  (0.00,0.04) | Moderate quality |
| 6608 | 132 | 57.02 |  | 91.55 | 0.01  (0.00,0.02) | High quality |
| Method Subgroups analysis | | | | | | |
| 7089 | 307 | 46.18 | 0.47 | 96.60 | 0.02  (0.01,0.05) | Agar dilution |
| 3740 | 101 | 48.26 |  | 85.67 | 0.00  (0.00,0.01) | E-test |
| 359 | 8 | 5.56 |  | - | 0.01  (0.00,0.08) | Broth dilution |

| Number of isolates | Number of Resistance isolate | % weight | Heterogeneity between groups (P-Value) | Heterogeneity | Poled resistance rate | Moxifloxacin (CLSI) |
| --- | --- | --- | --- | --- | --- | --- |
| 11484 | 3912 | 100 | 0.00 | 93.89 | 0.32  (0.25,0.40) | Overall |
| Year Subgroups analysis | | | | | | |
| 4636 | 1904 | 42.39 | 0.508 | 99.05 | 0.36  (0.22,0.51) | 1992-2014 |
| 6850 | 2008 | 57.61 |  | 97.26 | 0.30  (0.23,0.37) | 2015-2019 |
| Continent Subgroups analysis | | | | | | |
| 2386 | 920 | 24.68 | 0.000 | 99.30 | 0.28  (0.08,0.53) | Europe |
| 5293 | 1939 | 28.67 |  | 98.31 | 0.44  (0.33,0.55) | North America |
| 3016 | 942 | 34.15 |  | 94.37 | 0.33  (0.25,0.40) | Asia |
| 660 | 35 | 7.58 |  | 85.35 | 0.07  (0.01,0.14) | Oceania |
| 54 | 4 | 3.02 |  | - | 0.04  (0.00,0.13) | South America |
| 77 | 72 | 1.90 |  | - | 0.94  (0.85,0.98) | Africa |
| Quality Subgroups analysis | | | | | | |
| 50 | 27 | 1.86 | 0.014 | - | 0.54  (0.39,0.68) | Low quality |
| 4644 | 1767 | 45.11 |  | 98.88 | 0.35  (0.22,0.49) | Moderate quality |
| 6792 | 2118 | 53.03 |  | 98.00 | 0.30  (0.22,0.38) | High quality |
| Method Subgroups analysis | | | | | | |
| 7373 | 2332 | 57.57 | 0.543 | 98.02 | 0.29  (0.22,0.37) | Agar dilution |
| 139 | 40 | 40.49 |  | 98.91 | 0.38  (0.23,0.54) | E-test |
| 3974 | 1540 | 1.93 |  | - | 0.29  (0.21,0.37) | Broth dilution |

| Number of isolates | Number of Resistance isolate | % weight | Heterogeneity between groups (P-Value) | Heterogeneity | Poled resistance rate | Meropenem (CLSI) |
| --- | --- | --- | --- | --- | --- | --- |
| 2756 | 20 | 100 | 0.00 | 71.49 | 0.00  (0.00,0.01) | Overall |
| Year Subgroups analysis | | | | | | |
| 1155 | 0 | 39.92 | 0.106 | 0.00 | 0.00  (0.00,0.00) | 2002-2014 |
| 1601 | 20 | 60.08 |  | 81.80 | 0.00  (0.00,0.02) | 2015-2019 |

| Number of isolates | Number of Resistance isolate | % weight | Heterogeneity between groups (P-Value) | Heterogeneity | Poled resistance rate | Piperacillin-tazobactam (CLSI) |
| --- | --- | --- | --- | --- | --- | --- |
| 3041 | 8 | 100 | 0.62 | 0.00 | 0.00  (0.00,0.00) | Overall |

| Number of isolates | Number of Resistance isolate | % weight | Heterogeneity between groups (P-Value) | Heterogeneity | Poled resistance rate | Clindamycin (CLSI) |
| --- | --- | --- | --- | --- | --- | --- |
| 19645 | 6685 | 100 | 0.00 | 97.50 | 0.59  (0.53,0.65) | Overall |
| Year Subgroups analysis | | | | | | |
| 4704 | 2442 | 44.92 | 0.96 | 96.92 | 0.59  (0.50,0.67) | 2002-2014 |
| 6723 | 4243 | 55.08 |  | 97.66 | 0.59  (0.50,0.67) | 2015-2019 |
| Continent Subgroups analysis | | | | | | |
| 2585 | 1357 | 26.72 | 0.000 | 97.40 | 0.47  (0.34,0.60) | Europe |
| 4256 | 2069 | 20.87 |  | 98.00 | 0.47  (0.36,0.59) | North America |
| 3862 | 2766 | 41.75 |  | 94.95 | 0.72  (0.65,0.78) | Asia |
| 569 | 436 | 4.77 |  | - | 0.53  (0.19,0.86) | Oceania |
| 73 | 35 | 3.29 |  | - | 0.59  (0.19, 0.94) | South America |
| 82 | 22 | 2.61 |  | - | 0.25  (0.16, 0.36) | Central America |
|  |  | 0 |  | - | - | Africa |
| Quality Subgroups analysis | | | | | | |
| 70 | 12 | 2.95 | 0.000 | - | 0.17  (0.09,0.27) | Low quality |
| 3545 | 1866 | 40.76 |  | 97.43 | 0.57  (0.46,0.68) | Moderate quality |
| 7812 | 4807 | 56.29 |  | 97.52 | 0.63  (0.55,0.70) | High quality |
| Method Subgroups analysis | | | | | | |
| 6541 | 4158 | 48.68 | 0.020 | 97.57 | 0.64  (0.56,0.72) | Agar dilution |
| 4655 | 2373 | 48.06 |  | 97.10 | 0.53  (0.43,0.62) | E-test |
| 139 | 115 | 3.26 |  | - | 0.68  (0.53,0.65) | Broth dilution |

| Number of isolates | Number of Resistance isolate | % weight | Heterogeneity between groups (P-Value) | Heterogeneity | Poled resistance rate | Ciprofloxacin (CLSI) |
| --- | --- | --- | --- | --- | --- | --- |
| 4339 | 3356 | 100 | 0.00 | 99.12 | 0.95  (0.85,1.00) | Overall |
| Year Subgroups analysis | | | | | | |
| 1570 | 1569 | 57.00 | 0.001 | 0.00 | 1.00  (1.00,1.00) | 1992-2014 |
| 2769 | 1787 | 43.00 |  | 99.44 | 0.79  (0.54,0.97) | 2015-2019 |
| Continent Subgroups analysis | | | | | | |
| 947 | 908 | 32.30 | 0.000 | 97.43 | 0.96  (0.82,1.00) | Europe |
| 1305 | 600 | 17.85 |  | 99.64 | 0.94  (0.40,1.00) | North America |
| 2001 | 1806 | 40.25 |  | 97.28 | 0.96  (0.89,1.00) | Asia |
|  |  | 0 |  | - | - | Oceania |
| 0 | 0 | 2.87 |  | - | 1.00  (0.40, 1.00) | South America |
| 82 | 38 | 6.73 |  | - | 0.47  (0.36, 0.59) | Central America |
|  |  | 0 |  | - | - | Africa |
| Quality Subgroups analysis | | | | | | |
| 20 | 20 | 3.49 | 0.495 | - | 1.00  (0.83,1.00) | Low quality |
| 2213 | 1426 | 42.48 |  | 99.54 | 0.89  (0.59,1.00) | Moderate quality |
| 2106 | 1910 | 54.04 |  | 96.16 | 0.98  (0.93,1.00) | High quality |
| Method Subgroups analysis | | | | | | |
| 1791 | 1056 | 32.86 | 0.998 | 99.49 | 0.94  (0.66,1.00) | Agar dilution |
| 2548 | 2300 | 67.14 |  | 97.82 | 0.95  (0.87,1.00) | E-test |
|  |  | - |  | - | - | Broth dilution |

| Number of isolates | Number of Resistance isolate | % weight | Heterogeneity between groups (P-Value) | Heterogeneity | Poled resistance rate | Tetracycline (CLSI) |
| --- | --- | --- | --- | --- | --- | --- |
| 4861 | 886 | 100 | 0.020 | 97.50 | 0.59  (0.53,0.65) | Overall |
| Year Subgroups analysis | | | | | | |
| 1415 | 198 | 39.21 | 0.26 | 96.94 | 0.15  (0.06,0.28) | 1992-2014 |
| 3446 | 688 | 60.79 |  | 97.18 | 0.23  (0.15,0.33) | 2015-2019 |
| Continent Subgroups analysis | | | | | | |
| 1153 | 163 | 32.31 | 0.000 | 97.09 | 0.16  (0.05,0.31) | Europe |
| 1308 | 55 | 12.37 |  | 68.86 | 0.04  (0.02,0.08) | North America |
| 2281 | 626 | 49.07 |  | 95.41 | 0.26  (0.17,0.35) | Asia |
| 119 | 42 | 6.25 |  | - | 0.34  (0.26,0.43) | Oceania |
|  |  | 0 |  | - | - | South America |
|  |  | 0 |  | - | - | Central America |
|  |  | 0 |  | - | - | Africa |
| Quality Subgroups analysis | | | | | | |
| 70 | 32 | 5.99 | 0.01 | - | 0.40  (0.29,0.52) | Low quality |
| 1749 | 275 | 38.22 |  | 96.99 | 0.16  (0.07,0.28) | Moderate quality |
| 3042 | 579 | 55.79 |  | 97.36 | 0.22  (0.13,0.32) | High quality |
| Method Subgroups analysis | | | | | | |
| 436 | 1837 | 52.13 | 0.22 | 96.31 | 0.22  (0.13,0.33) | Agar dilution |
| 2932 | 439 | 44.59 |  | 97.72 | 0.18  (0.09,0.29) | E-test |
| 92 | 11 | 3.28 |  | - | 0.12  (0.06,0.20) | Broth dilution |

| Number of isolates | Number of Resistance isolate | % weight | Heterogeneity between groups (P-Value) | Heterogeneity | Poled resistance rate | Amoxicillin-Clavulanate () |
| --- | --- | --- | --- | --- | --- | --- |
| 2803 | 4 | 100 | 0.06 | 45.40 | 0.00  (0.00,0.00) | Overall |

| Number of isolates | Number of Resistance isolate | % weight | Heterogeneity between groups (P-Value) | Heterogeneity | Poled resistance rate | Ceftriaxone () |
| --- | --- | --- | --- | --- | --- | --- |
| 3476 | 1289 | 100 | 0.00 | 99.05 | 0.47  (0.29,0.65) | Overall |

**EUCAST**

| Number of isolates | Number of Resistance isolate | % weight | Heterogeneity between groups (P-Value) | Heterogeneity | Poled resistance rate | Rifampin (EUCAST) |
| --- | --- | --- | --- | --- | --- | --- |
| 1861 | 787 | 100 | 0.00 | 97.69 | 0.37  (0.18,0.58) | Overall |

| Number of isolates | Number of Resistance isolate | % weight | Heterogeneity between groups (P-Value) | Heterogeneity | Poled resistance rate | Moxifloxacin (EUCAST) |
| --- | --- | --- | --- | --- | --- | --- |
| 2809 | 929 | 100 | 0.00 | 98.68 | 0.49  (0.30,0.67) | Overall |

| Number of isolates | Number of Resistance isolate | % weight | Heterogeneity between groups (P-Value) | Heterogeneity | Poled resistance rate | Tigecycline  () |
| --- | --- | --- | --- | --- | --- | --- |
| 2375 | 39 | 100 | 0.00 | 83.53 | 0.01  (0.00,0.03) | Overall |
